# Maternal Anxiety and Depression From Pregnancy to Postpartum: Associations With Infants' Cortical Functional Connectivity at 1 Month

**DOI:** 10.1111/infa.70135

**Published:** 2026-11-03

**Authors:** Zayna K. Mian, Laura Carnevali, Borja Blanco, Maria Rozhko, Matthew Weatherhead, Liam H. Collins‐Jones, Sarah Lloyd‐Fox, Mark H. Johnson, The PIPKIN Study Team

**Affiliations:** ^1^ Department of Psychology University of Cambridge Cambridge UK; ^2^ Milner Therapeutics Institute University of Cambridge Cambridge UK

**Keywords:** functional connectivity, HD‐DOT, maternal anxiety, maternal depression

## Abstract

Maternal anxiety and depression have been associated with alterations in functional connectivity (FC) in infancy, a period in which rapid brain development and functional network organization have been linked to longer term socioemotional outcomes. Investigating maternal anxiety and depression within the same sample is important, given that they frequently co‐occur and may show distinct associations with infant brain development. A longitudinal approach is key to understanding whether and how changes in maternal mental health across the perinatal period relate to infant brain development. We examined associations of subclinical prenatal and postnatal maternal depression and anxiety, measured via questionnaires at 32 weeks of gestation and 1 month postpartum, with infants' FC at 1 month of age. Task‐free FC data were collected during natural sleep using High‐Density Diffuse Optical Tomography (HD‐DOT) in the home. No associations were observed between maternal anxiety symptoms and FC, and neither postnatal depression nor anxiety alone predicted infant FC. Lower maternal prenatal depression scores were associated with stronger FC in the infants' frontal network (*n* = 39). In contrast, greater increases in maternal depressive symptoms from the prenatal to postnatal periods were linked to increased frontal FC, particularly when mothers had higher prenatal depression levels (*n* = 28). Most symptom changes were small in magnitude and did not cross clinical thresholds, suggesting largely subclinical and transient mood shifts. We speculate that while early prenatal exposure may signal vulnerability in specific neural circuits, the subsequent increase in symptoms could trigger compensatory or accelerated development in the same networks. Replication with larger, more diverse, samples and longitudinal research is needed to determine whether these subtle early fluctuations in maternal symptoms are associated with longer‐term trajectories of infant brain development beyond the perinatal window.

## 1 Introduction

Maternal mental health, particularly anxiety and depression has been consistently associated with differences in infant brain development (Blanchett et al. 2023; Buss et al. 2010; Bijsterbosch 2017; Dean et al. 2018; Donnici et al. 2021; Dufford, Salzwedel, et al. 2021; Qiu et al. 2015; Soe et al. 2016; S. Wang et al. 2022). Depression is characterized by low mood and fatigue, while anxiety involves excessive worry and physiological arousal (American Psychiatr and ic Association 2013). Although global prevalence estimates of maternal postnatal depression and anxiety are around 17.22% and 15.2%, respectively (Dennis et al. 2017; Z. Wang et al. 2021), subclinical symptoms are more widespread and often go unrecognized. Previous studies have suggested that even mild maternal symptoms are related to infant development (Goodman and Tully 2008; Skotheim et al. 2013). Maternal anxiety and depression have been associated with variations in brain structure in children, such as reduced grey matter density in specific brain regions (Buss et al. 2010), as well as with differences in functional connectivity (FC) (Bijsterbosch 2017; Qiu et al. 2015), defined as the temporal correlation between neural signals from different brain regions. They have also been linked to difficulties in infants' socio‐emotional development (Dean et al. 2018; Feldman et al. 2009) with associations persisting into childhood and adolescence (A. Rogers et al. 2020). For example, subclinical depressive symptoms in mothers have been associated with lower infant responsiveness to social cues (Skotheim et al. 2013) and differences in white matter in frontal regions (Dean et al. 2018). Together, these findings highlight the importance of considering maternal mental health across a continuum of symptom severity, as effects on infant development may extend beyond clinically significant symptoms and diagnostic categories.

The timing and persistence of maternal anxiety and depression may also be important for understanding infant outcomes. Prenatal depression strongly predicts postnatal depression (Underwood et al. 2016), which is linked to poorer psychomotor and cognitive development and increased risk for conduct problems in toddlerhood (Cornish et al. 2005; Netsi et al. 2018). Prenatal anxiety is also associated with negative socioemotional outcomes, with evidence that the postnatal environment may mediate these associations (Nolvi et al. 2023; Rees et al. 2019). These associations have been proposed to involve biological pathways (e.g., fetal brain structure, stress reactivity; Aktar et al. 2019) and environmental pathways (e.g., caregiver‐infant interactions, attachment and parental sensitivity; Newman et al. 2017; Wu et al. 2024). Postnatal maternal sensitivity and social support may promote resilience and help mitigate the adverse effects of poor maternal mental health on infant development (Frigerio and Nazzari 2021; Takács et al. 2021). However, the relative contributions of prenatal and postnatal maternal symptoms, and the mechanisms underlying their associations to infant development remain unclear. Most studies focus on infants older than six months and overlook perinatal changes in maternal mental health, which may differentially relate to infant outcomes, underscoring the need for longitudinal research (Yatziv et al. 2021). Given the frequent co‐occurrence of anxiety and depression, it is also essential to investigate both their individual and combined associations with infant neurodevelopment (Deutsch et al. 2022; Field et al. 2010).

Early infancy, particularly the first few months postpartum, is characterized by rapid brain development, neural reorganization and the establishment of structural and functional brain networks (Dean et al. 2018; Gilmore et al. 2018), known to relate to later cognitive and behavioral outcomes (C. E. Rogers et al. 2017; Tooley et al. 2024; Yu et al. 2022). Functional connectivity (FC), which captures the temporal relationship between neurophysiological signals from different brain regions, provides insight into emerging functional brain organization (Bijsterbosch 2017). Task‐free FC is particularly suited to studying early functional brain development in very young infants who are frequently asleep during neuroimaging, enabling the examination of intrinsic network activity without task‐related confounds (Blanco et al. 2022; Carnevali et al. 2026; Gonzalez‐Castillo and Bandettini 2018).

Sensory networks like the primary sensorimotor and auditory networks develop early in infancy, followed by higher‐order frontal networks involved in attention and executive function (Doria et al. 2010; Gao, Alcauter, Smith, et al. 2015). Across early infancy, FC shifts from short‐range to long‐range connections, reflecting increased network integration, regional specialization and the emergence of large‐scale networks like the default mode network (DMN) and frontoparietal network (Fransson et al. 2011; Gao et al. 2009; Hu et al. 2022; Thomason et al. 2013). These networks are crucial for higher‐order functions such as executive function and emotional regulation (Fan et al. 2021; Ravi et al. 2023). Individual differences in FC during early infancy are linked to affect, cognition, and behavior, and vary with environmental factors including maternal mental health and socioeconomic status (Bruchhage et al. 2020; Kelsey et al. 2021; Gao, Alcauter, Elton, et al. 2015; Pozzi et al. 2021).

Given that individual differences in early FC have been related to environmental factors, maternal mental health may be particularly relevant to infant brain development. Prenatal maternal depression has been associated with stronger FC between the amygdala and temporal/insular regions involved in emotional regulation and memory in six‐month‐old infants of mothers from a non‐clinical sample (Qiu et al. 2015), resembling patterns observed in clinically depressed adults (Ramasubbu et al. 2014). Similarly, clinical prenatal depression has been associated with weaker FC in frontoparietal and temporal areas in newborns (S. Wang et al. 2022). In non‐clinical samples, high levels of prenatal anxiety have been associated with stronger amygdala FC and lower connectivity in temporal and parietal areas in children aged three to seven years (Donnici et al. 2021). These connectivity patterns have been associated with difficulties in emotional perception as well as externalizing behaviors (Mukherjee et al. 2012; Pagliaccio et al. 2015).

Postnatal maternal anxiety has likewise been associated with differences in FC in offspring, even in non‐clinical populations. For instance, 14‐month‐olds of mothers with higher postnatal anxiety showed lower FC between the left amygdala and right ACC (Dufford, Salzwedel, et al. 2021). Increases in maternal symptoms from pregnancy to postpartum were associated with greater right frontal FC and frontal asymmetry at six months (Soe et al. 2016), suggesting that changes in maternal symptoms across the perinatal period may be relevant to frontal brain function. Collectively, these studies indicate that maternal anxiety and depression, even at subclinical levels, are associated with FC in both subcortical regions, such as the amygdala, and cortical regions, such as temporal, parietal, and prefrontal areas. This body of work points to the perinatal period as a potentially important window during which maternal mood may be related to infant FC.

Despite this growing evidence, several important gaps remain. First, few studies have examined both anxiety and depression simultaneously within the same sample across the perinatal period, limiting our understanding of whether they show distinct or overlapping associations with infant FC (Deutsch et al. 2022). For example, Blanchett et al. (2023) found that maternal psychiatric symptoms, rather than genetic factors, were associated with stronger FC in the frontoparietal and ventral attention networks (Blanchett et al. 2023). However, by grouping together anxiety, depression, and other mental health conditions, the study precluded insight into the specific contributions of each. Second, much of the existing literature focuses on clinically significant symptoms or treats maternal mental health categorically, potentially overlooking associations with mild or subclinical symptoms. Framing maternal mental health as a continuum is crucial for understanding the subtle associations between mild or subclinical symptoms and infant development. Third, relatively few studies have considered changes in maternal symptoms across both prenatal and postnatal periods, despite evidence that symptom trajectories may be relevant to infant FC. Thus, longitudinal studies that examine both prenatal and postnatal influences at an early age, and that consider maternal mental health symptoms on a continuum rather than as a binary construct, are essential for understanding their unique and cumulative associations with infant brain development (Na et al. 2023).

The current exploratory study, focusing on a non‐clinical sample, aims to address these gaps by examining associations between prenatal and postnatal anxiety and depression scores and infant FC at 1 month of age, using High‐Density Diffuse Optical Tomography (HD‐DOT), a non‐invasive neuroimaging tool that measures cortical hemodynamic activity. As discussed above, previous research has linked maternal anxiety and depression to differences in FC in both subcortical regions and frontal networks. Here, we focused on frontal, auditory, and sensorimotor networks (subcortical regions could not be reliably accessed with this technology). Frontal networks may be especially sensitive to maternal mental health symptoms due to their protracted and experience‐dependent postnatal development (Hodel 2018). While sensorimotor and auditory networks also develop rapidly during prenatal and early postnatal periods, they reach relatively adult‐like topologies soon after birth, exhibiting strong within‐network connectivity as well as high consistency in connectivity patterns across individuals (Desrosiers et al. 2024; Fransson et al. 2011; Gao, Alcauter, Smith, et al. 2015). Because auditory and sensorimotor networks develop earlier and show relatively stable connectivity patterns soon after birth, we treat them as control networks in our study. In contrast to the stability of primary sensory networks, FC in frontal regions is characterized by reduced segregation at birth and high interindividual variability (Carnevali et al. 2026; Gao, Alcauter, Smith, et al. 2015; Turk et al. 2019), and their developmental trajectories have been suggested to be more susceptible to environmental influences (Carnevali et al. 2026). This motivated us to explore whether higher maternal anxiety and depression scores, at both prenatal and postnatal timepoints, will show stronger associations with FC strength in the frontal brain network, compared to the auditory and sensorimotor networks at 1 month.

The use of a longitudinal study design also allowed us to explore the relative contributions of prenatal and postnatal maternal anxiety and depression scores, and whether symptom change across timepoints is associated with greater differences in infant FC at 1 month of age. We anticipated that, postnatal maternal anxiety and depression scores may show a stronger association with infant frontal FC strength than prenatal scores. This is because frontal areas remain relatively less mature than sensorimotor, visual and temporal areas, suggesting they may retain greater sensitivity to postnatal environmental experiences during a period of rapid functional reorganisation (Desrosiers et al. 2024; Turk et al. 2019). Maternal symptoms may be especially salient postnatally as they are linked to differences in caregiving behaviors such as maternal sensitivity, which has in turn been associated with postnatal FC development (Bernard et al. 2018; Ilyka et al. 2021; Stern et al. 2024; Carnevali et al. 2026). The quality of the postnatal caregiving environment may also moderate the associations between prenatal stress and infant neurodevelopment (Nolvi et al. 2023). Nevertheless, associations between prenatal maternal symptoms and FC have also been reported in the literature, with some studies suggesting that symptom trajectories, rather than the timing of symptoms alone, may be more closely related to FC patterns (Severo et al. 2023; Soe et al. 2016). We therefore consider the relative contributions of prenatal and postnatal maternal anxiety and depression symptoms to be an open empirical question. Thus, we examine whether infants exposed to elevated maternal mental health symptoms at both timepoints differ in FC from those exposed at a single timepoint, which may help provide preliminary insights into whether persistent maternal symptoms are associated with infants' FC, a measure of functional brain development.

In summary, the study aims to:

1. Characterize maternal anxiety and depression symptoms across prenatal and postnatal timepoints in a non‐clinical sample.
2. Characterize infant FC in frontal, auditory and sensorimotor networks at 1 month of age.
3. Test the primary hypothesis that higher maternal anxiety and depression scores, measured prenatally and postnatally, will show stronger associations with infant FC at 1 month of age in the frontal network compared to the auditory and sensorimotor networks.
4. Examine two exploratory aims: first, whether associations between maternal anxiety and depression and infant FC differ between prenatal and postnatal timepoints, and second, whether changes in maternal anxiety and depression symptoms from pregnancy to postpartum were associated with infant FC, including whether these associations varied by network and baseline prenatal symptom scores.

## 2 Methods

### 2.1 Participants

Participants were drawn from the Perinatal Imaging in Partnership with Families (PIPKIN) study, a UK based longitudinal project examining how early life experiences and family environment relate to infant development (https://www.pipkinstudy.com; Clackson et al. 2025). Ethical approval was received from the Cambridge Psychology Research Ethics Committee (Application no.: PRE.2020.123) and the East Midlands—Nottingham 1 Research Ethics Committee, on behalf of the NHS Health Research Authority (IRAS project ID: 295027, REC ref. 22/EM/0004). Informed written consent was obtained from parents. The current study included a subsample of *n* = 74 of the [blinded for peer review] cohort. Of these, *n* = 53 parents completed the Hospital Anxiety and Depression Scale (HADS) questionnaire prenatally and *n* = 57 postnatally. An additional *n* = 3 prenatal and *n* = 7 postnatal questionnaires were collected but were incomplete and excluded. Valid FC data were available for *n* = 56 participants, with an additional *n* = 18 datasets excluded during preprocessing (cap displacement *n* = 4; low channel count *n* = 2; insufficient recording duration *n* = 12). Final sample sizes varied by analysis, depending on availability of valid FC data and complete HADS responses (Figure 1). Further details regarding the demographic characteristics of the sample, including maternal education, household income, and ethnicity are included in the Supporting Information S1: Table S1 and S2 SM.

**FIGURE 1:**
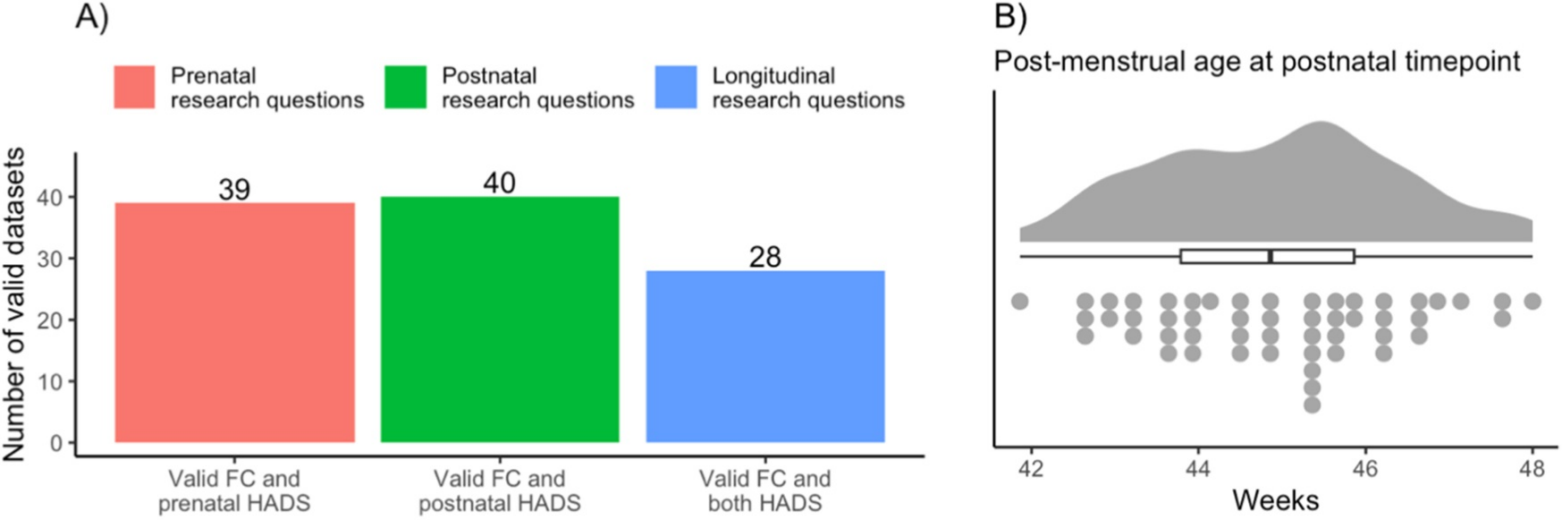
(A) Summary of the sample sizes included in the analyses corresponding to prenatal (*n* = 39; 17 Female, 22 Male provided prenatal HADS and valid FC at 1 month), postnatal (*n* = 40 16F, 24M provided postnatal HADS data and valid FC at 1 month), and longitudinal (*n* = 28 12F, 16M provided HADS data at both prenatal and postnatal timepoints as well as valid FC at 1 month) research questions. (B) Distribution of participants' postmenstrual age (PMA) in weeks at the time of the postnatal scan (*M* = 44.93, SD = 1.47, range: 41.86–48 weeks). Chronological age at the postnatal timepoint for participants with valid FC and at least one completed HADS ranged between 26 and 47 days after birth (*M* = 36.49, SD = 4.83).

### 2.2 Data Acquisition

#### 2.2.1 Questionnaires

Maternal anxiety and depression were assessed using the HADS questionnaire, administered at 32 weeks' gestation at the Rosie Hospital (Cambridge, UK) and at 1 month postpartum during a study home visit. Mothers completed the questionnaire either online or on an iPad during the visit (Supporting Information S1: Table S3 and Figure S1). The HADS questionnaire takes approximately 2–5 minutes to complete includes two subscales: anxiety and depression, each with 7 items rated on a 4‐point Likert scale (range: 0–21). Scores ≤ 7 are considered within the normal range, 8–10 indicate borderline symptoms, > 11 reflect clinical levels. Higher scores reflect greater symptoms severity (Bjelland et al. 2002; Zigmond and Snaith 1983).

#### 2.2.2 HD‐DOT Acquisition

Task‐free FC data were collected during home visits at 1 month using HD‐DOT, while infants were sleeping and being held by an experimenter. The cap was positioned with both ears visible and the top edge above the eyebrows. We used a HD‐DOT system (LUMO, Gowerlabs Ltd, UK; https://www.gowerlabs.co.uk/lumo), comprising 12 tiles (each with 4 photodiode detectors and 3 dual‐wavelength sources) covering frontal and temporal regions. Signal quality was monitored in real time, with a target of at least three motion‐free 100‐s segments, up to 600 seconds (mean recording time: 16.2 min, SD = 5.9; mean clean time: 11.4 min, SD = 3.7; *n* = 51).

We aimed for ∼10 minutes of clean data, as longer acquisitions improve FC stability, sensitivity, and precision (Van Dijk et al. 2010; J. Wang et al. 2017; Paranawithana et al. 2023). Extended durations also allow for the capture of low‐frequency fluctuations (e.g., 0.01 Hz = 100 s cycles), key to FC analysis. Participants with < 250 seconds of usable data were excluded, consistent with prior infant fNIRS FC studies (Bulgarelli et al. 2020; Lee et al. 2020; Uchitel et al. 2023; Blanco et al. 2021). One participant was excluded due to insufficient channel retention (Supporting Information S1: Figure S2). Cap placement was evaluated from multi‐angle session photos using a three‐tier index relative to the forehead, informing tilt correction during spatial registration (Supporting Information S1: Figures S6 and S7). Full methods are in the Supporting Information S1: Figures S3–S5.

### 2.3 Data Processing

Data processing was performed in MATLAB using DOTHUB and Homer2 toolboxes, as well as Toast++ (Huppert et al. 2009; Schweiger and Arridge 2014). The pipeline entailed data preprocessing, image reconstruction, cortical parcellation and FC computation, described below. Full function names, parameter settings and signal diagnostic plots are included in the Supplementary Materials.

#### 2.3.1 Preprocessing of HD‐DOT Data

Preprocessing focused on identifying data segments ≥ 100 seconds with minimal motion artifacts. Motion artifacts were identified in short separation channels (< 12 mm). Channels where > 90% of the recording was identified as motion were discarded. Time points were retained if > 90% of short‐separation channels were motion‐free, and continuous segments ≥ 100 seconds were extracted. To maximize the duration of clean segments, and to include additional segments, brief motion artifacts (< 2 sec) were retained and corrected using wavelet denoising (Patel et al. 2014). Low‐amplitude channels were also excluded (dRange = [1e‐4, 2.5]). Within each segment, a 60‐s window with maximal channel retention was automatically selected, using a signal‐to‐noise ratio of 12.5 (Frijia et al. 2021; Carnevali et al. 2024). Intensity data were converted to optical density, then to HbO and HbR concentrations. Short‐separation regression (SSR) was applied to attenuate the impact of extracerebral confounds (Vidal‐Rosas et al. 2021). Bandpass filtering was performed using linear regression model: sine and cosine functions modeled physiological signals (> 0.08 Hz) (Caballero‐Gaudes and Reynolds 2017), while Legendre polynomials accounted for fluctuations at very low frequencies (Blanco et al. 2021). Data were converted back to optical density and down sampled to 1/10th of its original frequency to optimize file size. Final datasets per participant were created by concatenating the normalized signal of each clean segment, retaining only channels that were consistently preserved across all clean segments.

#### 2.3.2 Image Reconstruction

We selected a neonatal head model (37.9 cm, 44.28 weeks) from the Collins‐Jones et al. (2021) database to match our cohort's (37.9 cm, 44.3 weeks). Optode and landmark coordinates were projected onto the model for individual‐level reconstruction. Sensitivity profiles (Jacobians) were computed via finite element modeling in Toast++ (Schweiger and Arridge 2014). HbO/HbR concentration changes were reconstructed using zeroth‐order Tikhonov regularization (hyperparameter = 0.01). For each participant, a binary sensitivity mask was created by thresholding the normalized Jacobians at 10% of the maximum. These were summed across participants, and a group‐level mask was created by retaining nodes sensitive in ≥ 80% of participants (Uchitel et al. 2023). This mask was applied to all further analyses to constrain results to reliably sampled cortical regions.

#### 2.3.3 Cortical Parcellation

Cortical parcellation was used to reduce dimensionality. The M‐CRIB neonatal atlas (Alexander et al. 2019) was processed with the developing Human Connectome Project pipeline (Makropoulos et al. 2018) and non‐linearly registered to the head model using ANTs (Avants et al. 2008). Labels from 10 atlas subjects were combined via joint label fusion to create a 70‐region parcellation. This was intersected with the group‐level sensitivity mask, retaining parcels where at least 50% of nodes were within the sensitive field of view, yielding 25 cortical parcels for FC analysis (Figure 2).

**FIGURE 2:**
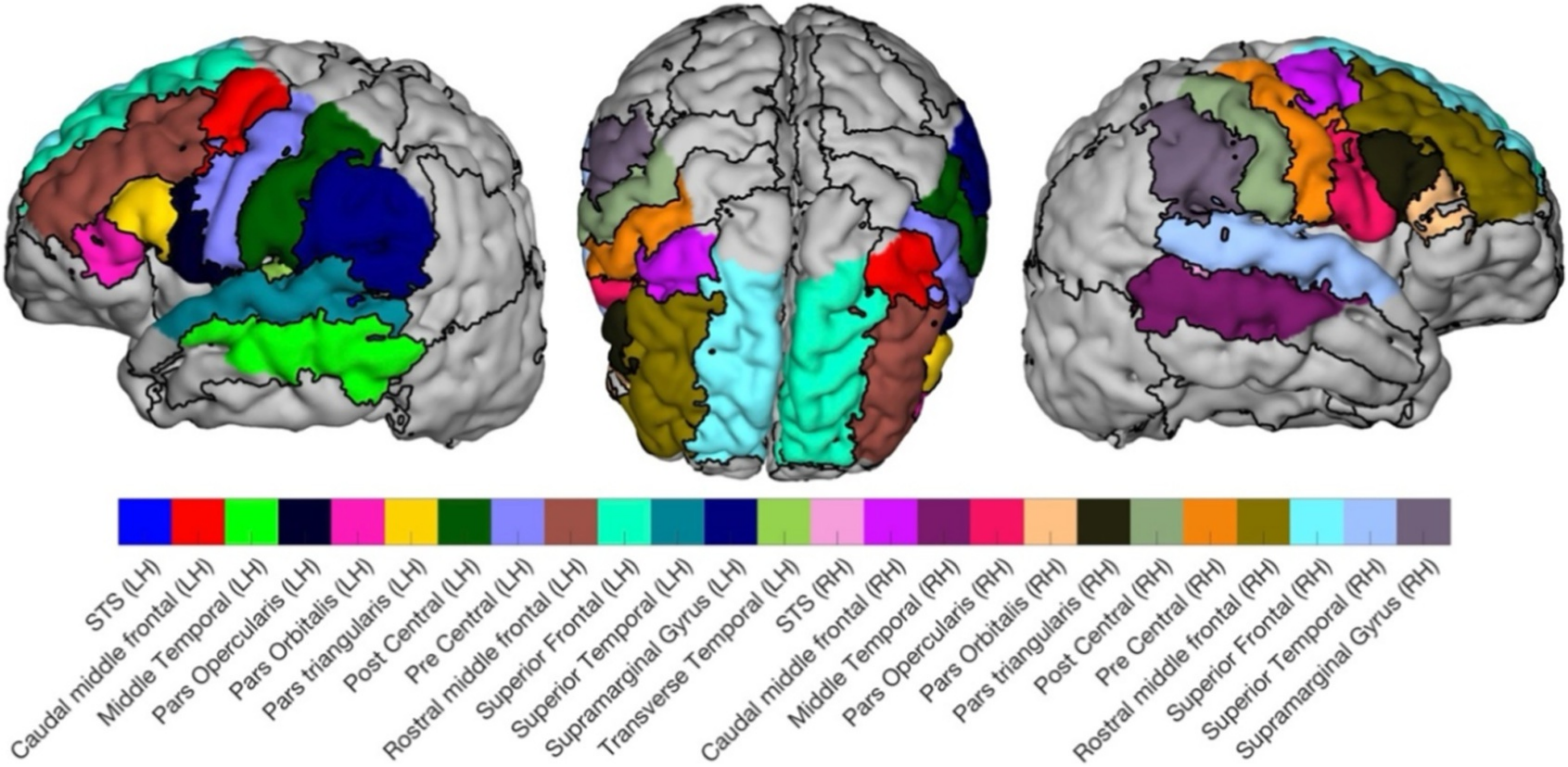
Cortical parcellation.

### 2.4 Network Selection and FC Computation

HbO and HbR signals were extracted for each participant at nodes within each parcel. For both chromophores, the node time series within a parcel were averaged to yield a single representative time course. Parcel‐wise FC matrices were then computed by calculating Pearson correlations between all parcel pairs and applying Fisher z‐transformation for group‐level analyses. This produced a 25 × 25 FC matrix per participant, for HbO and HbR. A seed‐based correlation analysis was conducted using parcels from the 25‐region atlas. The frontal network, defined by seeds in the bilateral superior frontal gyrus (Hacker et al. 2013; Smyser et al. 2010), was the primary network of interest. Control networks included the sensorimotor and auditory networks, with seeds placed bilaterally in the precentral (Gao, Alcauter, Elton, et al. 2015) and superior temporal (Ciarrusta et al. 2020) parcels, respectively.

To define network‐level masks, we identified significant connections across the full sample (*n* = 51) using one‐sample *t*‐tests on z‐transformed values, with FDR correction at *p* < 0.001. A z‐threshold of 0.3095 (*r* = 0.3) was further applied to retain only the strongest connections. These group‐level network masks were then applied to each participant's z‐transformed FC matrices, and mean z‐scores were computed for the frontal, auditory, and sensorimotor networks (see Figure 3 and Supporting Information S1: Figure S14 for seed‐based correlation maps for HbO and HbR). Scores were calculated separately by hemisphere, then averaged per seed, per network, for both HbO and HbR, and used in statistical analyses.

**FIGURE 3:**
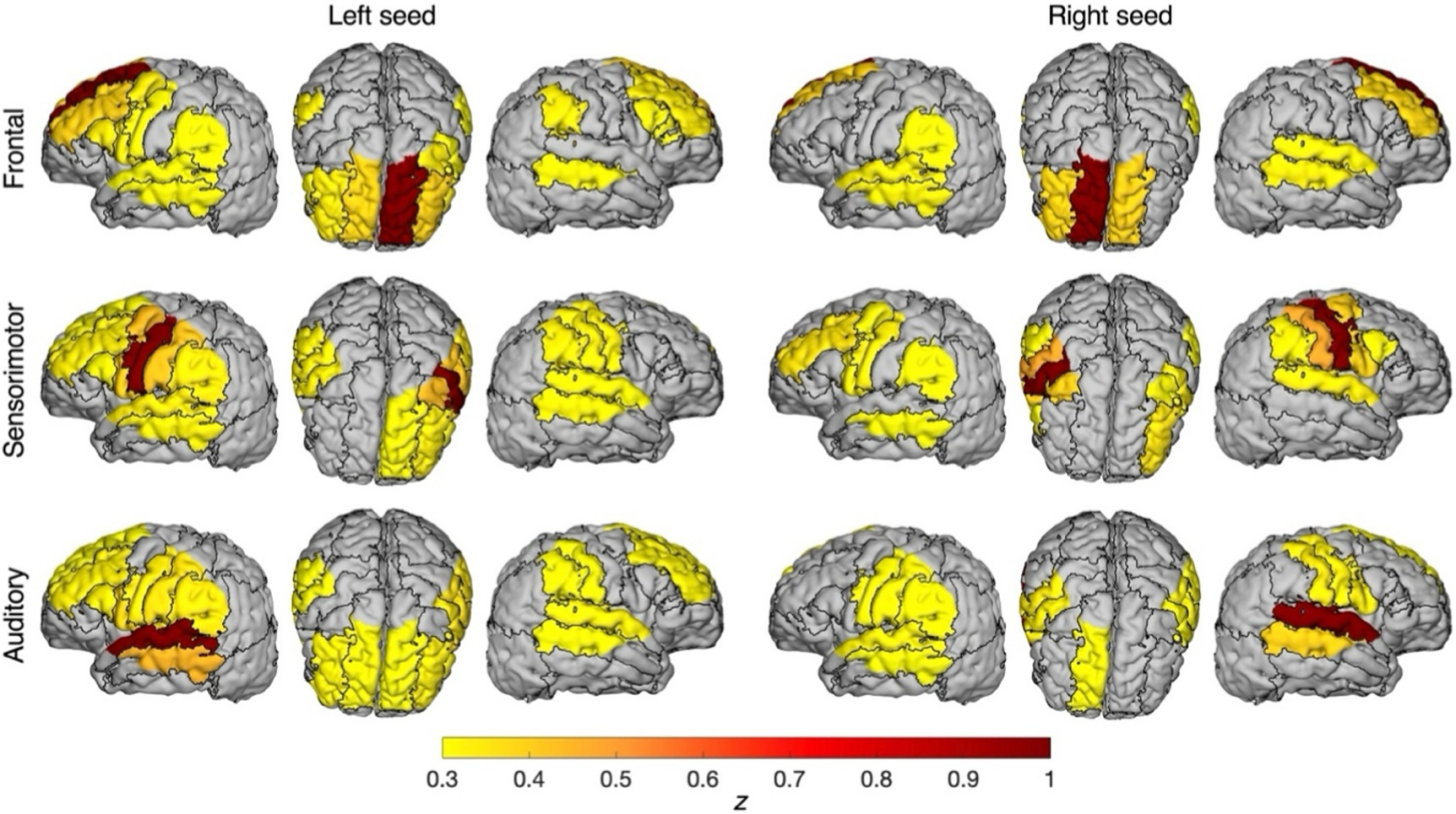
Group‐level seed‐based FC maps for the frontal (top), sensorimotor (middle) and auditory (bottom) networks at 1 month of age. The maps show Fischer‐Z transformed correlation values for each network, separately for left and right hemisphere seeds.

Our primary analyses were conducted on the HbO chromophore consistent with current reporting practices in the infant HD‐DOT and fNIRS literature, thus facilitating direct comparison with existing studies (Kinder et al. 2022). Nevertheless, parallel analyses for HbR were run and are provided in the supplementary materials in accordance with best practice recommendations for reporting fNIRS results (Yücel et al. 2021).

### 2.5 Statistical Analysis

Statistical analyses were performed using R (R Core Team 2013). For each dependent variable, linear mixed‐effect models were built using the *lmer()* function from the lme4 package (Bates et al. 2015) and compared using *Akaike Information Criterion (AIC)* via the *compare_performance()* function (Lüdecke et al. 2021). The model with the lowest AIC was considered the best‐fitting (Hooper et al. 2008), with AIC weights used to quantify relative model plausibility (Wagenmakers and Farrell 2004). We examined the best model's fixed‐effect estimates, *p*‐values, and *R* ^2^ values (marginal and conditional). Mixed models accounted for fixed effects (e.g., HADS scores, network, timepoint, slopes; see Table 1) and included participant as a random effect to capture individual variability (Baayen et al. 2008; Gelman and Hill 2006).

**TABLE 1:** List of dependent and independent variables.

Dependent variables	FC strength: Averaged *z* transformed HbO values (HbR reported in SM)
Independent variables	Network: 3 level factor‐ frontal, auditory and sensorimotor
	Timepoint: 2 level factor‐ prenatal, postnatal
	Age: Numeric variable indicating postmenstrual age (PMA) in weeks at which FC was obtained
	Anxiety: Numerical value between 0–21 for prenatal and postnatal timepoint separately
	Depression: Numerical value between 0–21 for prenatal and postnatal timepoint separately
	Slope anxiety: Linear rate of change in anxiety scores between prenatal and postnatal timepoints per individual.
	Slope depression: Linear rate of change in depression scores between prenatal and postnatal timepoints per individual.

We first assessed whether infant age, or its interaction with network, was related to FC by comparing three linear mixed‐effects models: a null model, a model with age as a fixed effect, and one with an age‐by‐network interaction. While the interaction model had better AIC and conditional *R* ^2^, age‐related effects were not statistically significant. Therefore, age was excluded from subsequent models to maintain parsimony.

At each timepoint (prenatal and postnatal), we built increasingly complex models to test main and interaction effects of anxiety, depression, and network on FC. Longitudinal models included individual slopes of maternal symptoms, baseline prenatal scores, and their interactions (including three‐way interactions with network) to assess how symptom changes relate to FC across networks, as part of our exploratory analyses. All models included random intercepts for participants. Full model details and comparisons are in the Supporting Information S1.

## 3 Results

### 3.1 Descriptive Statistics

#### 3.1.1 Maternal Anxiety and Depression Across the Perinatal Period

Across both prenatal and postnatal timepoints, most mothers scored within the normal range for both anxiety and depression on the HADS. Descriptive statistics for anxiety and depression scores across timepoints are reported in Table 2 and shown in Figure 4.

**TABLE 2:** Mean anxiety and depression scores.

Scale	Timepoint	*n*	Mean	Median	SD	Range
Anxiety	Prenatal	39	6.28	6	3.85	0–18
	Postnatal	40	5.42	5	3.58	0–19
Depression	Prenatal	39	4.92	5	2.96	0–12
	Postnatal	40	5.03	4	3.42	0–13

**FIGURE 4:**
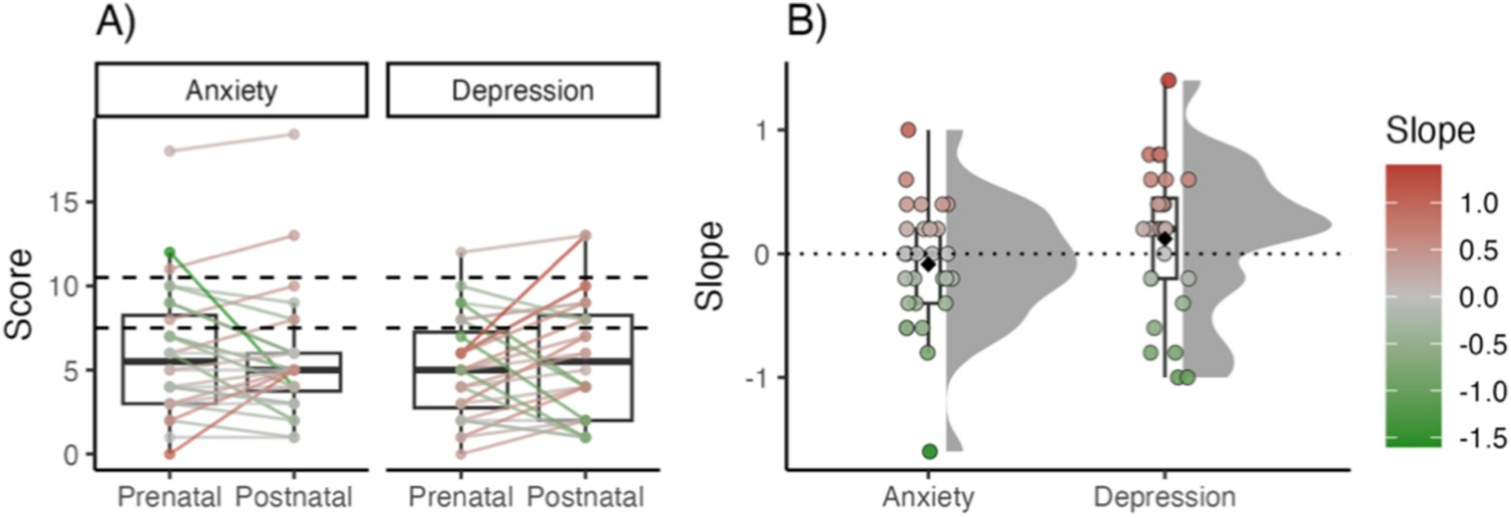
Maternal HADS anxiety and depression scores across prenatal and postnatal assessments. (Panel A) depicts anxiety and depression scores by scale and timepoint. Dashed lines represent cut‐offs between normal (0–7), borderline (8–10) and clinical (> 11) symptoms. Color coding refers to the slope, with positive numbers leaning toward red and negative numbers leaning toward green. (Panel B) shows distributions of slopes, for anxiety and depression scores separately, from prenatal to postnatal timepoints. Dotted line at Slope = 0 means no change between prenatal and postnatal, while positive numbers indicate an increase in symptoms and negative numbers indicate a decrease. Diamonds represents the mean.

#### 3.1.2 Infant Functional Connectivity at 1 Month

The group‐level seed maps in Figure 3 display the strength of correlations between seed regions and other brain regions across the frontal, sensorimotor and auditory networks at 1 month of age, with seeds placed in both left and right hemispheres.

### 3.2 Primary Analyses

Primary analyses were conducted using HbO, with parallel analyses also run on HbR. Main results are reported below for HbO across the three periods, while full details on model comparison, diagnostic checks and full results on HbR are reported in the Supporting Information S1: Sections 8 and 9, Figures S8–S13; Tables S5–S7. Associations between maternal symptoms and infant FC were primarily observed for depression rather than anxiety. Prenatal maternal depression was negatively associated with frontal FC. Postnatal depression was not associated with FC. The results for HbR largely converged with HbO as described in the Supporting Information S1: Section 10, Table S8.

#### 3.2.1 Prenatal Maternal Symptoms and Infant Functional Connectivity

The selected prenatal model included an interaction between Anxiety and Network and Depression and Network and demonstrated the best fit (AIC = 65.991; AIC weight = 0.671), with the model explaining 46.6% of the total variance. The model summary revealed a significant interaction between Network and Depression (*F* (2, 72) = 4.16, *p* = 0.019). Higher Prenatal Depression scores were found to be significantly associated with lower FC in the frontal network (*β* = −0.04, 95% CI [−0.09, −0.001], *t* (106) = −2.02, *p* = 0.046). Neither the main effect of Prenatal Anxiety nor the interaction between Anxiety and Network emerged as significant, as shown in Table 3. A visualization of the effect is in Figure 5, Panel A.

**TABLE 3:** Fixed effects results for the selected prenatal model (HbO).

Term	*β*	95% CI	SE	*t* (106)	*p*
Fixed effects					
Intercept	0.42	[0.19, 0.66]	0.117	3.62	< 0.001
Anxiety	−0.01	[−0.04, 0.02]	0.015	−0.84	0.404
Network [frontal]	0.40	[0.15, 0.66]	0.129	3.11	0.003
Network [auditory]	−0.07	[−0.33, 0.18]	0.129	−0.57	0.571
Depression	0.01	[−0.03, 0.05]	0.019	0.49	0.624
Anxiety × network [frontal]	−0.001	[−0.03, 0.03]	0.016	−0.09	0.928
Anxiety × network [auditory]	−0.001	[−0.03, 0.03]	0.016	−0.06	0.955
Depression × network [frontal]	−0.04	[−0.09, < 0.001]	0.021	−2.02	0.046
Depression × network [auditory]	0.02	[−0.03, 0.06]	0.021	0.77	0.441
Random effects					
ID (intercept)—SD	0.20	[0.14, 0.29]	0.04		
Residual—SD	0.25	[0.22, 0.30]	0.02		
*N* (observations)	117				
*N* (groups)	39				

*Note:* 95% CIs and *p*‐values were computed using a Wald *t*‐distribution approximation. The reference level for Network is Sensorimotor. Model formula: z_score ∼ Anxiety × Network + Depression × Network + (1|ID).

Abbreviations: *β*, unstandardized regression coefficient; SD, standard deviation; SE, standard error.

**FIGURE 5:**
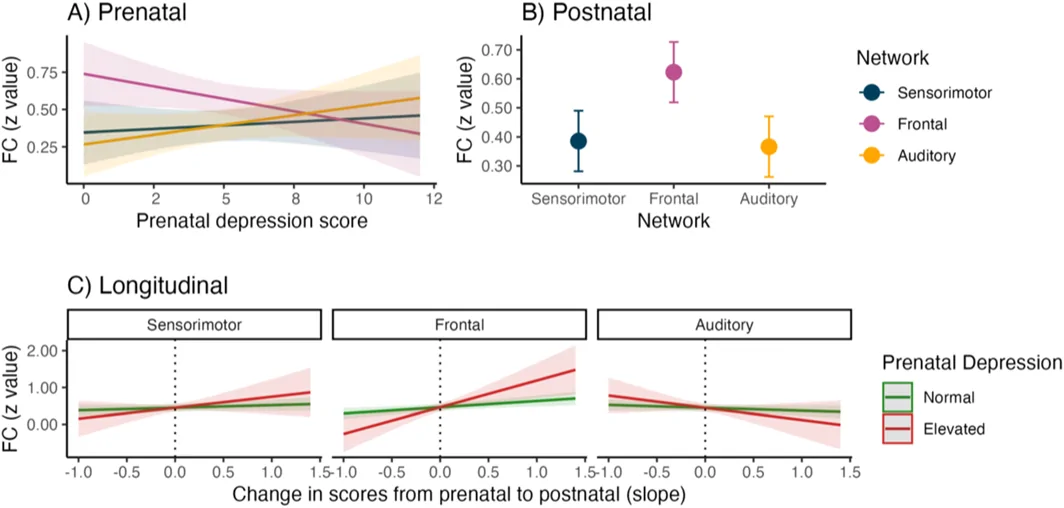
(Panel A) shows the relationship between prenatal depression scores and infant FC at 1 month for each network. (Panel B) depicts the main effect of network on FC at 1 month. (Panel C) illustrates the effect of longitudinal change in maternal depression levels on infant FC at 1 month, separately by network and stratified by prenatal depression levels. The clustering of prenatal depression into normal and elevated levels is for visualization purposes only, as the variable is treated as continuous in our analyses.

#### 3.2.2 Postnatal Maternal Symptoms and Infant Functional Connectivity

The selected postnatal model included main effects of Anxiety, Depression and Network and demonstrated the best fit (AIC = 72.964; AIC weight = 0.964), with the model explaining 41.8% of the total variance. A significant main effect of network was observed (*F* (2, 78) = 11.17, *p* < 0.001), with infants showing significantly greater FC in the frontal network compared to the sensorimotor network (*β* = 0.24, 95% CI [0.12, 0.36], *t* (113) = 3.92, *p* < 0.001). The main effects of Postnatal Anxiety and Postnatal Depression were not significant, as shown in Table 4. A visualization of the effect is in Figure 5, Panel B.

**TABLE 4:** Fixed effects results for the selected postnatal model (HbO).

Term	*β*	95% CI	SE	*t* (113)	*p*
Fixed effects					
Intercept	0.42	[0.24, 0.59]	0.088	4.72	< 0.001
Anxiety	−0.01	[−0.04, 0.01]	0.012	−0.90	0.376
Depression	0.006	[−0.02, 0.03]	0.013	0.47	0.643
Network [frontal]	0.24	[0.12, 0.36]	0.061	3.92	< 0.001
Network [auditory]	−0.02	[−0.14, 0.10]	0.061	−0.32	0.749
Random effects					
ID (intercept)—SD	0.19	[0.13, 0.29]	0.04		
Residual—SD	0.27	[0.23, 0.32]	0.02		
*N* (observations)	120				
*N* (groups)	40				

*Note:* 95% CIs and *p*‐values computed using a Wald *t*‐distribution approximation. Reference level for Network is Sensorimotor. Model formula: z_score ∼ Anxiety + Depression + Network + (1|ID).

Abbreviations: *β*, unstandardized regression coefficient; SD, standard deviation; SE, standard error.

With regard to the exploratory comparison between prenatal and postnatal timepoints, the results indicate a depression‐related association with FC in the prenatal model. However, no significant effects were observed for postnatal depression or for maternal anxiety across prenatal and postnatal timepoints.

### 3.3 Exploratory Analyses

#### 3.3.1 Longitudinal Associations Between Maternal Symptoms and Infant Functional Connectivity

The set of exploratory longitudinal models examined whether individual‐level changes in maternal anxiety and depression from prenatal to postnatal timepoints associated with infant FC at 1 month across different brain networks. Longitudinally, increases in maternal depression from prenatal to postnatal timepoints were associated with stronger frontal FC, particularly at higher levels of prenatal depression.

The selected longitudinal model included a three‐way interaction between Network, Change in Anxiety and Prenatal Anxiety as well as a three‐way interaction between Network, Change in Depression and Prenatal Depression, and explained 49.9% of the total variance in FC. A significant interaction was observed between Network × Slope of Depression × Prenatal Depression level (*F* (3, 37.94) = 6.12, *p* = 0.002). In the frontal network specifically, greater increases in maternal depression (more positive slopes) were significantly associated with stronger FC at 1 month (*β* = 0.05, 95% CI [0.02, 0.09], *t* (75) = 3.05, *p* = 0.003). In contrast, the interaction involving Network × Change in Anxiety × Prenatal Anxiety was not statistically significant, as reported in Table 5.

**TABLE 5:** Fixed effects results for the selected longitudinal model (HbO).

Term	*β*	95% CI	SE	*t* (75)	*p*
Fixed effects					
Intercept	0.45	[0.35, 0.55]	0.052	8.63	< 0.001
Network × Δ_Anxiety Prenatal_Anxiety					
Sensorimotor	−0.004	[−0.03, 0.03]	0.015	−0.24	0.812
Frontal	−0.028	[−0.06, 0.001]	0.015	−1.93	0.059
Auditory	0.000	[−0.03, 0.03]	0.015	0.03	0.976
Network × Δ_Depression × Prenatal_Depression					
Sensorimotor	0.022	[−0.01, 0.06]	0.018	1.25	0.216
Frontal	0.054	[0.02, 0.09]	0.018	3.05	0.003
Auditory	−0.025	[−0.06, 0.01]	0.018	−1.40	0.168
Random effects					
ID (intercept)—SD	0.22	[0.14, 0.33]	0.05		
Residual—SD	0.26	[0.22, 0.32]	0.03		
*N* (observations)	84				
*N* (groups)	28				

*Note:* 95% CIs and *p*‐values computed using a Wald *t*‐distribution approximation. Reference level for Network is Sensorimotor. Δ_Anxiety/Δ_Depression = change in anxiety/depression scores from prenatal to postnatal timepoints. Prenatal_Anxiety/Prenatal_Depression = HADS subscale scores at 32 weeks gestation. Model formula: z_score ∼ Network: Δ_Anxiety: Prenatal_Anxiety + Network: Δ_Depression: Prenatal_Depression + (1|ID).

Abbreviations: *β*, unstandardized regression coefficient; SD, standard deviation; SE, standard error.

#### 3.3.2 Prenatal to Postnatal Symptom Change

To contextualize the longitudinal depression finding, we examined the association between prenatal depression and subsequent change in symptoms as an exploratory post‐hoc analysis. Prenatal depression was negatively correlated with change in depression scores (*r* = −0.26). Moreover, most mothers with lower prenatal symptoms (15 of 21) exhibited an increase in depression symptoms postnatally, whereas among those with higher prenatal symptoms, some showed an increase (*n* = 3) and others a decrease (*n* = 4) (Figure 6, Panel A). Importantly, among mothers with lower prenatal symptoms who exhibited changes, the majority experienced only small fluctuations of ≤ 2 points in their depression scores postnatally (37.5% showed a 1‐point change, 25% showed a 2‐point change; see Supporting Information S1: Table S4 SM for details). Additionally, only a small subset of those with positive slopes (*n* = 5; see Figure 6, Panel B) transitioned from normal to elevated levels of symptoms (crossing the threshold score of 7). These findings suggest that the changes observed in this group largely reflect minor symptom fluctuations that remain within the subclinical range (Figure 6, Panel B).

**FIGURE 6:**
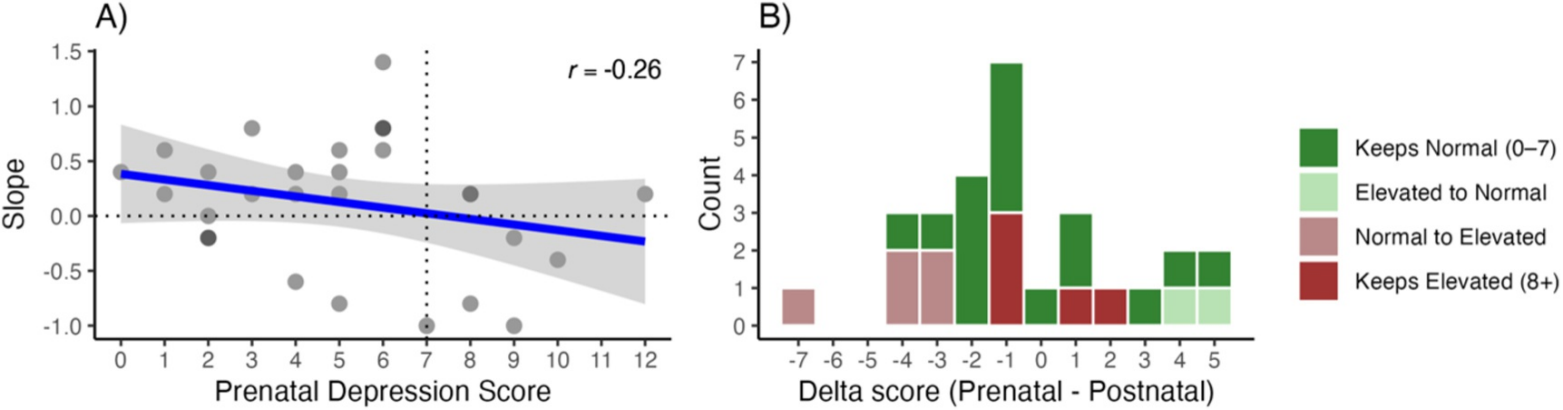
(Panel A) Correlation between prenatal depression scores and the change in depression from prenatal to postnatal periods. The horizontal dotted line represents no change in symptoms (slope = 0), while the vertical dotted line marks the threshold between normal and borderline scores based on the HADS scoring system. Darker dots indicate overlapping data points (i.e., multiple mothers reported the same score and change) resulting in darker shading. (Panel B) shows binned changes in depression scores (prenatal–postnatal) by 1‐point increments in both positive and negative directions. Bars are color‐coded by symptom status change categories, indicating whether mothers' symptoms remained within the normal range, shifted from normal to elevated, from elevated to normal, or stayed elevated, with a threshold score of 7 marking the boundary.

Importantly, infants of mothers whose prenatal levels of depression were within the normal range would not show differences in FC patterns as a function of changes in maternal symptoms postnatally (i.e., flat trend of green line in Panel C of Figure 5—central pane), while this would not be the case for infants whose mothers who scored higher in depression prenatally (i.e., positive trend of red line in Panel C of Figure 5—central pane).

## 4 Discussion

This study explored associations between maternal anxiety and depression, measured both prenatally and postnatally, on infant FC at 1 month. Symptoms were assessed dimensionally to capture variability beyond clinical thresholds. We found preliminary evidence that lower prenatal depression scores were associated with stronger infant frontal FC. Our exploratory analyses revealed that increases in maternal depressive symptoms from prenatal to postnatal periods were associated with stronger frontal FC, particularly in infants whose mothers' prenatal depression levels were higher. We speculate that this pattern may reflect an adaptive reorganization associated with changing maternal depression symptoms during the perinatal period, although this cannot be tested given the modest sample size and the single infant neuroimaging timepoint. While an overall increase in depression is observed postnatally at the group level, most mothers experienced only small fluctuations that did not result in a change from normal to elevated depression scores. Rather than clinically meaningful deterioration, these changes suggest normal variability in this population adjusting to an important change in their familial structure and routines. In fact, given the challenging postpartum period, such small symptom fluctuations are not unexpected (Phua et al. 2020) and may represent transient mood variability related to adjusting to having a young baby. We speculate that for most mothers experiencing these minor changes, symptoms are likely to decrease again over subsequent months, which will be evaluated in future follow‐ups.

In contrast to the observed depression‐related associations, changes in maternal anxiety symptoms across the perinatal period were not associated with infant FC, and neither postnatal maternal depression nor anxiety alone predicted FC strength in the current sample. Given the small sample in the current study, the lack of significant associations with anxiety do not rule out an association. Nevertheless, these preliminary findings suggest that perinatal maternal depression may show distinct, network‐specific associations with FC depending on the timing, magnitude and persistence of symptoms. We interpret our results in light of their consistency with prior research, then consider underlying neurobiological and adaptive mechanisms. We further discuss pathways through which maternal mental health may be associated with infant FC, even in subclinical populations.

Our preliminary finding of stronger frontal FC in infants of mothers with lower prenatal depressive symptoms aligns with previous reports linking higher maternal depression scores to lower frontal FC. For example, prior studies found lower FC in the frontal lobe (Na et al. 2023), prefrontal‐limbic circuits (Posner et al. 2016) and between frontoparietal regions (S. Wang et al. 2022) in infants exposed to prenatal depression. These findings are consistent with the possibility of network‐specific vulnerability of frontal networks to maternal depressive symptoms. Developmentally, frontal areas play an important role in emotional regulation, attention and executive functions (Hodel 2018), and differences in frontal connectivity, particularly with limbic regions, are thought to be closely linked to the risk of psychopathology (Braun and Bock 2011). The lack of a significant association between prenatal anxiety and FC may reflect differences in the infant brain correlates of maternal anxiety and depression, consistent with previous studies indicating that maternal prenatal anxiety is particularly associated with differences in FC in limbic regions in infants (Donnici et al. 2021; Dufford et al. 2021; Rifkin‐Graboi et al. 2015), such as the amygdala. This region was not accessible in the current study using HD‐DOT. It is also possible, however, that our non‐significant anxiety findings were due to insufficient statistical power, rather than the absence of an association, given our small longitudinal sample size (*n* = 28). This warrants replication in larger samples to clarify the apparent specificity of associations between maternal depression versus anxiety with infant frontal FC.

Beyond prenatal associations, we found that increasing depressive symptoms from the prenatal to postnatal period were associated with stronger frontal FC in our exploratory model, especially among those infants whose mothers reported higher symptoms prenatally. This interaction effect should be interpreted as preliminary given the limited statistical power to reliably estimate interaction effects. Nevertheless, one speculative interpretation is that this pattern may be consistent with adaptive reorganization in response to changes in maternal depression symptoms during the perinatal period, with stronger frontal FC reflecting a compensatory response to escalating postnatal adversity. Supporting this interpretation, Posner et al. (2016) reported stronger inverse FC (i.e., more negative correlations) between amygdala‐prefrontal circuits in infants of mothers with prenatal depression, suggesting functional adaptation to a potentially more challenging postnatal environment. A framework that potentially aligns with these findings is the stress acceleration hypothesis, which proposes that early adversity can expedite the development of brain circuits involved in emotion regulation to better equip the infant for more demanding conditions (Callaghan and Tottenham 2016). This interpretation is consistent with other studies linking early life stress to accelerated neural maturation in newborns (DiPietro et al. 2010), although our study did not measure neural developmental change. Since we assessed FC at a single timepoint only, longitudinal neuroimaging data are required to evaluate whether increasing maternal depressive symptoms during the perinatal period are prospectively associated with changes in infant frontal FC over time.

In contrast, for those with lower prenatal symptoms, postnatal changes in depression were not significantly associated with infant FC. Notably, among mothers with initially low symptoms who exhibited postnatal increases, the majority experienced only minor changes (typically 1–2 points) that did not reach the clinical threshold. These findings suggest that the observed changes likely reflect transient, subclinical fluctuations in mood, rather than sustained or clinically meaningful deterioration. Given the challenges of the early postpartum period—including disruptions to sleep, routine, and caregiving demands—such mood variability is not unexpected. It may instead represent a normative psychological adjustment to early parenthood and may be temporary and self‐resolving for many individuals. Follow‐up studies are needed to assess the trajectory of maternal symptoms beyond the early postnatal period, to determine whether these small increases persist or remit over time, and how they may associate with long‐term FC outcomes.

Together, the associations between perinatal depression and frontal FC may potentially reflect different but complementary processes related to infant brain development, although this study did not directly assess any underlying mechanisms. While one interpretation is that early prenatal exposure may be related to vulnerability in specific neural circuits, the subsequent increase in maternal symptoms could trigger compensatory or accelerated development in the same networks. This suggests that prenatal and longitudinal patterns of maternal mental health may jointly relate to infant FC, possibly through interacting risk‐exposure and adaptation processes, operating across both biological and caregiving pathways. Biologically, maternal psychological distress has been proposed to affect fetal brain development via alterations in the HPA axis, placental gene expression, inflammation, and epigenetic regulation, which are relevant for the developing frontal cortex (Wu et al. 2024). Fetal imaging studies have shown that maternal distress is associated with differences in frontoparietal and striatal connectivity in utero, and that supportive interpersonal environments may buffer these associations, suggesting potential opportunities for early intervention (Thomason et al. 2021). These processes unfold within a broader caregiving context, as maternal depression has been linked to less responsive caregiving—characterized by reduced emotional attunement, verbal input, and cognitive stimulation, which may limit early experiences that support the development of self‐regulation and learning (Severo et al. 2023). These behavioral and biological processes may interact, highlighting the complexity of associations between perinatal maternal depression depressive symptoms and infant neurodevelopment.

Our preliminary findings emphasize the need to consider maternal symptoms over time, rather than focusing on isolated prenatal or postnatal periods. Soe et al. (2016) similarly found that increases in maternal depressive symptoms from prenatal to postnatal timepoints—but not either timepoint alone—were associated with stronger frontal connectivity and asymmetry in infants. This dynamic perspective offers greater insight into how early neurodevelopment adapts to evolving maternal mental health contexts. Our findings are also broadly consistent with predictive adaptive models, which propose that outcomes depend on the relative match or mismatch between prenatal exposures and postnatal environments (Posner et al. 2016; Sandman et al. 2012). From this perspective, increasing depressive symptoms may signal an environmental mismatch, which could be related to the stronger frontal FC observed here, although this would need to be tested in larger longitudinal neuroimaging studies. The lack of associations for postnatal symptoms alone in our study further highlights the importance of considering the roles of cumulative exposure and symptom trajectories on infant FC.

## 5 Strengths and Limitations

A central limitation of the study is the relatively modest analytic sample sizes across analyses (prenatal *n* = 39, postnatal *n* = 40, longitudinal *n* = 28). These sample sizes are broadly consistent with other HD‐DOT studies in early infancy (Collins‐Jones et al. 2024; Frijia et al. 2021; Uchitel et al. 2023), where data attrition and data quality constraints are a prevalent and well documented challenge (Baek et al. 2023). Nevertheless, they still constrain the statistical power and interpretability of the current findings. Lower statistical power may increase the likelihood of Type II errors and can impact the precision and reliability of statistically significant effects, including the ability to reliably estimate higher‐order interaction effects, which typically require greater sample sizes and statistical power to detect (Button et al. 2013; Heo and Leon 2010). Therefore, the non‐significant associations observed for anxiety or postnatal symptoms alone should not be interpreted as the absence of an association, while the significant effects observed for maternal depression and FC should be interpreted with some caution, warranting further replication in larger samples. This is especially true for the longitudinal sample, which was considerably reduced due to questionnaire data not being available at both prenatal and postnatal timepoints. Therefore, the findings should be interpreted as preliminary, with replication in larger, more adequately powered samples needed to draw more definitive conclusions regarding associations between perinatal maternal mental health and infant FC.

Another limitation was the use of generic optode locations for spatial registration based on cap placement categories which were designed to approximate head tilt. Previous research in adults has shown that generic optode locations fail to account for variations in cap positioning across participants, with individualized optode registration methods producing better spatial precision with HD‐DOT (Srinivasan et al. 2024). Applying these methods in infants is challenging due to their limited tolerance for long setup times and to difficulties in obtaining high‐quality images. We also adopted a parcel‐based approach to define FC networks, simplifying analysis and reducing the computational demands of processing HbO and HbR matrices across thousands of grey matter nodes. However, this means potentially masking subtle or subregional connectivity patterns, as well as restricting our ability to ensure full coverage across networks of interest; for example, coverage across the full sensorimotor strip was limited, with only partial coverage of both the precentral and postcentral parcels. Finer‐grained, node‐based methods and a finer grained parcellation scheme would help preserve spatial specificity and potentially improve the accuracy of findings (Eickhoff et al. 2018; Schaefer et al. 2018).

Maternal mental health was assessed using HADS, which although well validated, was developed for clinical screening and may be less sensitive to subtle symptom variation in subclinical populations. Alternative measures validated for perinatal use, such as the Edinburgh Postnatal Depression Scale (EPDS) for depression or the State‐Trait Anxiety Inventory (STAI) for anxiety, may provide more sensitive estimates of symptom variability. Lastly, although this study examined prenatal and postnatal maternal mental health longitudinally, FC patterns also undergo rapid changes during the first month of life. Therefore, incorporating longitudinal FC measures in future studies can help better differentiate prenatal influences on FC from postnatal changes that may reflect increasing maternal depressive symptoms over time.

Despite these limitations, the study has several key strengths. The successful use of at‐home HD‐DOT acquisition in infants at 1 month of age represents an important advance in neuroimaging research, particularly in early infancy where MRI and other imaging methods are often impractical. The focus on a subclinical sample, which captures normative variation in maternal anxiety and depression, provides valuable insight into whether even mild symptoms relate to infants' functional brain connectivity during a critical window of early brain development. This approach may support the early identification of at‐risk infants, even when maternal symptoms do not reach clinical thresholds. Furthermore, the use of a longitudinal design allowed us to examine how changes in maternal symptoms over time are associated with differences in infant FC in early infancy, extending prior research that often focuses on a single perinatal timepoint or considers anxiety and depression in isolation. Since early FC has been linked to a range of cognitive, emotional and behavioral outcomes later in infancy and childhood (Bruchhage et al. 2020), understanding the associations between maternal mental health and infant FC is essential. These preliminary insights may help inform early identification of risk factors and contribute to a better understanding of how maternal psychological well‐being relates to infant FC, a key aspect of functional brain development.

## 6 Conclusions

Our preliminary findings are consistent with the view of maternal mental health as a key component of nurturing care. In this exploratory study, FC in the frontal network at 1 month was associated with maternal depressive symptoms, even within subclinical ranges. Although the findings need to be replicated in larger samples to draw more definitive conclusions regarding associations between maternal mental health and infants' frontal FC, the results highlight the potential relevance of the timing and trajectory of perinatal mental health symptoms in relation to infant FC. Given that frontal connectivity during infancy has been linked to later socio‐cognitive and emotional outcomes, early identification and support for maternal depressive symptoms, even at subclinical levels, during the perinatal period remain important for maternal well‐being, infant neurodevelopment and the broader caregiving environment. Integrating mental health screening into routine perinatal care and enhancing caregiver support may be promising ways to strengthen the caregiving environment during the first 1000 days.

## Author Contributions

**Zayna K. Mian:** conceptualization, formal analysis, writing – original draft, writing – review and editing. **Laura Carnevali:** conceptualization, formal analysis, writing – original draft, visualization, data curation, investigation, supervision, writing – review and editing. **Borja Blanco:** conceptualization, formal analysis, writing – original draft, visualization, writing – review and editing, investigation, supervision, data curation. **Maria Rozhko:** investigation, writing – review and editing. **Matthew Weatherhead:** investigation, writing – review and editing. **Liam H. Collins‐Jones:** writing – review and editing, visualization. **Sarah Lloyd‐Fox:** conceptualization, supervision, resources, funding acquisition, writing – review and editing. **Mark H. Johnson:** conceptualization, supervision, funding acquisition, resources, writing – review and editing.

## Funding

B.B. and M.J. were supported by a Medical Research Council Programme Grant (MR/T003057/1). L.C. and S.L.F. were supported by a UKRI Future Leaders fellowship (MR/S018425/1).

## Ethics Statement

Ethical approval was received from the Cambridge Psychology Research Ethics Committee (Application no.: PRE.2020.123) and the East Midlands—Nottingham 1 Research Ethics Committee, on behalf of the NHS Health Research Authority (IRAS project ID: 295027, REC ref. 22/EM/0004).

## Conflicts of Interest

The authors declare no conflicts of interest.

## Supporting information


Supporting Information S1

## Data Availability

Data will be available upon request from the authors, subject to completion of the relevant data sharing agreements in accordance with ethical guidelines governing sensitive data.
