# Supplementary material for: Maternal Anxiety and Depression From Pregnancy to Postpartum: Associations With Infants' Cortical Functional Connectivity at 1 Month
Source: Infancy. 2026 Nov 3;31(6):e70135. doi: 10.1111/infa.70135 (PMC13731146; doi:10.1111/infa.70135)
Supplement: Supplementary file 1 [file INFA-31-0-s001.docx]

**SUPPLEMENTARY MATERIALS**

Table of Contents

[1.	Sample Demographics	1](#_Toc240282420)

[2.	Anxiety and depression by collection method	2](#_Toc240282421)

[3.	Details on exclusion of neuroimaging datasets	3](#_Toc240282422)

[4.	Detailed HD-DOT data processing	4](#_Toc240282423)

[a.	Preprocessing of HD-DOT data	4](#_Toc240282424)

[b.	Image Reconstruction	7](#_Toc240282425)

[c.	Cortical Parcellation	7](#_Toc240282426)

[5.	Cap positioning and three-tiered index for spatial registration	7](#_Toc240282427)

[6.	Changes in depression across categories	9](#_Toc240282428)

[7.	Model comparisons (HbO and HbR)	10](#_Toc240282429)

[8.	Model Diagnostics	16](#_Toc240282430)

[9.	Model Results for HbR	22](#_Toc240282431)

[10. Significant Effects Across HbO and HbR	24](#_Toc240282432)

# 1. **Sample Demographics**

**Table 1 SM.**

*Frequency and Percentage of Mother and Baby Ethnicity*

|  | **Category** | **n (%)** |
| --- | --- | --- |
| ***Mother Ethnicity*** | | |
|  | Asian or Asian British | 6 (12.2%) |
|  | Mixed or Multiple ethnic groups | 3 (6.1%) |
|  | Other ethnic group | 1 (2%) |
|  | White (British) | 32 (65.3%) |
|  | White (Other) | 7 (14.3%) |
| ***Baby Ethnicity*** | | |
|  | Asian or Asian British | 3 (6.1%) |
|  | Mixed or Multiple ethnic groups | 9 (18.4%) |
|  | Other ethnic group | 1 (2%) |
|  | White (British) | 32 (65.3%) |
|  | White (Other) | 4 (8.2%) |
|  | | |

*Note.* Percentages are based on participants with non-missing data.

**Table 2 SM.**

*Frequency and Percentage of Maternal Education and Monthly Household Income*

|  | **Category** | **n (%)** |
| --- | --- | --- |
| ***Mother Education*** | | |
|  | GCSEs (or education to 16 years) | 1 (2%) |
|  | Vocational Qualification | 2 (4.1%) |
|  | A Level (or education to 18 years) | 2 (4.1%) |
|  | Degree | 19 (38.8%) |
|  | Postgraduate qualification | 25 (51%) |
| ***Monthly Household Income (Pre-birth)*** | | |
|  | £2,000 – £2,600 | 4 (10%) |
|  | £2,600 – £3,600 | 1 (2.5%) |
|  | £3,600 – £4,600 | 15 (37.5%) |
|  | £4,600 – £5,600 | 7 (17.5%) |
|  | £5,600 – £6,600 | 4 (10%) |
|  | £6,600 – £8,000 | 2 (5%) |
|  | £8,000 – £10,000 | 3 (7.5%) |
|  | Do not wish to answer | 4 (10%) |
|  | | |

*Note.* Percentages are based on participants with non-missing data. Education categories are listed by qualification level.

# 2. **Anxiety and depression by collection method**

**Table 3 SM.**

*Summary statistics of HADS scores by collection method (online link for completion in own time vs iPad administered during session)*

| **Timepoint** | **Measure** | **Method** | **N** | **Mean** | **SD** | **Min** | **Max** |
| --- | --- | --- | --- | --- | --- | --- | --- |
| Prenatal | Anxiety | iPad | 9 | 7.11 | 5.46 | 0 | 18 |
|  |  | online | 30 | 6.03 | 3.31 | 1 | 13 |
|  | Depression | iPad | 9 | 5.44 | 2.92 | 1 | 10 |
|  |  | online | 30 | 4.77 | 3 | 0 | 12 |
| Postnatal | Anxiety | iPad | 27 | 5.78 | 3.94 | 0 | 19 |
|  |  | online | 13 | 4.69 | 2.69 | 1 | 10 |
|  | Depression | iPad | 27 | 5.07 | 3.06 | 0 | 13 |
|  |  | online | 13 | 4.92 | 4.21 | 1 | 13 |

**Figure 1 SM.** Distributions of HADS scores by collection method

# 3. **Details on exclusion of neuroimaging datasets**

**Figure 2 SM.** (A) Distribution of clean data duration (seconds) among participants with FC datasets available and HADS scores available at least for one timepoint (n = 64). Dotted line indicates the 250 sec threshold for inclusion. The one datapoint that is far from the others indicates one of the early babies tested, where we were working out for how long recording could go on, therefore for that participant we recorded for as much as they were happy to. (B) Distribution of retained channels per wavelength.

# 4. **Detailed HD-DOT data processing**

Data processing was performed in MATLAB using DOTHUB and Homer2 toolboxes, as well as Toast++ (Huppert et al. 2009; Schweiger & Arridge, 2014). The pipeline entailed data preprocessing, image reconstruction, cortical parcellation and FC computation. Each of these steps is described below.

# a. **Preprocessing of HD-DOT data**

Our pre-processing pipeline comprised three main steps: identifying clean data segments, pre-processing each segment, and concatenating the normalised signal across segments.

Step 1: Identifying clean data segments

Clean data segments that were at least 100 seconds long were first identified and extracted. In an initial assessment of the data, motion artifact detection was performed on short separation channels (<12mm) using the *hmrMotionArtifactByChannel* function with the following parameters: tMotion = 0.5, tMask = 5, STDEVthresh = 10, AMPthresh = 0.1. Parameters were selected based on Uchitel et al. (2023). Those channels where more than 90% of the recording was identified as motion were rejected. Then, for each time sample we computed the percentage of motion-free channels, to identify time points where more than 90% of short-distance channels were motion-free. From this, clean segments that were at least 100 seconds long were extracted. For each participant, each segment was inspected to assess the presence of cardiac pulse in the signal, and motion artifacts. Figure 2 shows representative signal diagnostics that were examined for each participant, including motion artifact detection output across short-separation channels over time and raw intensity signals.

**Figure 3 SM.** Signal Quality Diagnostics for a Representative Participant. (Top) Heat-map showing the output of motion artifact detection (using the *hmrMotionArtifactByChannel* function) across short-separation channels over time. Values of 1 (yellow) indicate clean, motion-free timepoints and values of 0 (red) indicate timepoints containing motion artifacts. (Bottom) Raw Intensity signal data across all channels over the same recording,

Step 2: Pre-processing each segment

Channels displaying low amplitude were first excluded using *enPruneChannels* function (dRange = [1e-4, 2.5]). To maximize the duration of clean segments, and to include additional segments, brief motion artifacts (less than 2 sec) were included and later corrected by means of wavelet denoising (Patel et al, 2014). For each clean segment, channel rejection was performed with a customization of *enPruneChannels* function which uses a 60-second sliding window to automatically select a segment with the highest channel retention based on its signal-to-noise ratio of 12.5 (Frijia et al., 2021; Carnevali et al., 2024). The resulting channel distribution and signal quality for a representative participant following this step are illustrated in Figure 3. Intensity data were then converted to optical density and oxy- and deoxyhaemoglobin (HbO and HbR) concentration changes using *hmrOD2Conc* function. Short-separation regression (SSR) was included, to attenuate the impact of extracerebral confounds (*DOTHUBhmrSSRegressionByChannel*, flagSSmethod = 2, rhoSDssThresh = 10, Vidal-Rosas et al., 2021), as shown in Figure 4. This was followed by bandpass filtering, using a linear regression model to remove very low frequency fluctuations (Caballero-Gaudes & Reynolds, 2017). Sine and cosine functions for frequencies above 0.08 Hz were used to remove the contribution of physiological noise sources (e.g. respiration and cardiac pulsation), and Legendre polynomials were included to account for fluctuations at very low frequencies (Blanco et al., 2021). After filtering, we converted concentration data back to optical density for image reconstruction using *hmrConc2OD* function and down sampled to 1/10^th^ of its frequency to optimize file size, allowing for separate files to be generated for each clean data segment. At this point, we used the *DOTHUBwritePREPRO* function to save a separate file for each preprocessed data segment.

Step 3: Concatenation across segments

After preprocessing all the clean segments, their normalized signal was concatenated for those channels that were consistently retained across data segments, generating a single dataset for each participant.

**Figure 4 SM.** Preprocessing quality diagnostics run for each clean segment (i.e. in this case first green segment in Figure 2 SM). (Top-left) Optode layout showing source (red) and detector (blue) positions on the scalp. (Top-centre) Histogram of channels by source-detector separation distance. (Top-right) Power spectral density of raw signals grouped by source-detector distance. (Bottom) Raw signal time courses for all channels across the recorded segment.

**Figure 5 SM.** Effect of Short Separation Regression (SSR) on fNIRS signal quality. The plot shows data before (top) and after (bottom) SSR is applied for a representative participant.

# b.  **Image Reconstruction**

A single head model was used for image reconstruction across all participants. This model was selected from the database provided by Collins-Jones et al. (2021), which includes a set of structural neonatal head models varying in head circumference and gestational age. To ensure anatomical relevance, we selected the head model whose combination of head circumference and gestational age most closely matched the average values observed in our sample (M = 37.97 cm, SD = 1.19, range: 34-41 cm). Specifically, the selected head model’s head circumference measured 37.9 cm, with a gestational age of 44.28 weeks. Individual-specific optode and landmark coordinates, as estimated by our optode registration method, were projected onto the head model and used for subject-level image reconstruction. The sensitivity profile (Jacobian matrix) for each wavelength was computed using the finite element method in Toast++ (Schweiger & Arridge, 2014). A zeroth-order Tikhonov regularised reconstruction was performed using a regularisation hyperparameter of 0.01. Reconstructed images of changes in HbO and HbR concentrations were derived at each time point for every node of the grey matter mesh. The code employed for preprocessing and reconstructing the HD-DOT data is available as part of the DOT-HUB toolbox at www.github.com/DOT-HUB. A group-level sensitivity mask was calculated by finding the sensitivity for each surface node at the participant level using the *DOTHUB_MakeGMSensitivityMap* function. A node was considered sensitive if it exhibited a sensitivity greater than 10% the maximum value of the normalized Jacobian. For each participant, a binary sensitivity mask was computed, which was then summed across participants, and any nodes which were sensitive in >80% of participants were included in the final group-level mask (Uchitel et al., 2023).

# c. **Cortical Parcellation**

A cortical parcellation approach was employed to reduce computational load during FC analysis. The M-CRIB neonatal atlas (Alexander et al., 2019), including 10 neonatal T2-weighted MRI scans, was processed using the developing Human Connectome Project (Makropoulos et al., 2018) pipeline to create brain masks and segment different tissue types. Then each atlas brain was aligned with the head model by matching the intensity distributions with the T2-weighted scans, after which each M-CRIB T2 volume was non-linearly registered to the head model using the ANTs toolbox (Avants et al., 2008). Following this, the labels from all 10 atlas subjects were combined using a joint label fusion approach, resulting in a cortical parcellation with 70 regions of interest. The group-level sensitivity mask was applied to the cortical parcellation, producing a map of parcels that the array was sensitive to. Parcels were included if the array was sensitive to at least 50% of the nodes belonging to that parcel. Once the sensitivity profile was applied to the parcellated cortex, 25 parcels remained within the field-of-view of our experiment.

# 5. **Cap positioning and three-tiered index for spatial registration**

The quality of cap positioning was noted based on pictures acquired during the session from different angles and used to exclude participants when the cap was displaced. Cap placement was considered correct when placed above infants’ eyebrows, with the tiles centred, ensuring coverage of frontal brain regions. Four participants were excluded from analysis due to poor cap placement or tiles not being centred. For participants with adequate cap positioning, a three-tiered index (Figure 4 SM) was used to classify the extent to which cap covered participant’s forehead. Tier 1: Cap placed just above infants’ eyebrows (ideal placement, n = 23). Tier 2: Cap placed in mid-frontal position (half of forehead visible, n = 19). Tier 3: Cap placed close to the hairline (more than half forehead visible, n = 9).

**Figure 6 SM.** A) Ideal placement: with cap placed just above infant’s eyebrows. B) Tier 2: Cap placed in mid-frontal position (half of forehead visible). C) Tier 3: Cap placed close to the hairline (more than half forehead visible). D) Poor cap placement: excluded from analyses.

The three-tiered index was later used for registering source-detector positions to the head model. A custom MATLAB function was developed to compensate for variations in HD-DOT cap placement across participants. This function applies a smooth, graded backward rotation to the headband layout by rotating optode positions around a common posterior anchor point and a fixed axis (the x-axis). The degree of rotation for each tile in the layout is proportional to its anterior-posterior position, with the most anterior tiles rotated by the maximum specified angle and posterior tiles remaining nearly fixed. This approach preserves the spatial geometry of the optode layout and maintains consistent within tile structure, ensuring that the headband configuration accurately reflects differences in headband placement while minimising distortion of the original layout. Similar methods have been used in previous studies using HD-DOT with infant participants (Collins-Jones et al., 2024, Speh et al., 2025), for which accurate and easy to use method for spatial registration of optode locations are not yet available. Based on the cap positioning categories, fixed rotations of 7.5 degrees (Tier 2) and 15 degrees (Tier 3) were applied to approximate cap tilt (Figure 5 SM).

**Figure 7 SM.** Cap positioning categories used to approximate tilt, with example figures. Three cap positions are shown on the infant head model: Tier 1 (neutral/original placement), Tier 2 (tilted by 7.5 degrees) and Tier 3 (tilted by 15 degrees).

# 6. **Changes in depression across categories**

**Table 4 SM.**

*Changes in depression symptoms from prenatal to postnatal timepoints across different status change categories.*

| **Change in depression from prenatal to postnatal** | **n** | **No change** | **1pt change** | **2pt change** | **>2pt change** |
| --- | --- | --- | --- | --- | --- |
| Keeps Normal (0–7) | 16 | 1 (6.2%) | 6 (37.5%) | 4 (25%) | 5 (31.2%) |
| Elevated to Normal | 2 | 0 | 0 | 0 | 2 (100%) |
| Normal to Elevated | 5 | 0 | 0 | 0 | 5 (100%) |
| Keeps Elevated (8+) | 5 | 0 | 4 (80%) | 1 (20%) | 0 |

Note. The table shows the number of mothers (n) in each category, along with counts and percentages (in parentheses) of mothers experiencing no change, 1-point change, 2-point change, or greater than 2-point change in depression scores.

# 7. **Model comparisons (HbO and HbR)**

| **Testing age effect alone on functional connectivity (HbO)** | | | | | | | | | | |
| --- | --- | --- | --- | --- | --- | --- | --- | --- | --- | --- |
| **Model** | **Formula** | **Description** | **AIC** | **AIC_wt** | **R2_conditional** | **R2_marginal** | **ICC** | **RMSE** | **Sigma** |  |
| M0 | z_score ~ 1 + (1\|ID) | Null model with random intercept only | 95.226 | 0.000 | 0.251 | 0.000 | 0.251 | 0.264 | 0.289 |  |
| M1 | z_score ~ Age + (1\|ID) | Main effect of age on FC | 97.244 | 0.000 | 0.259 | 0.000 | 0.259 | 0.262 | 0.289 |  |
| M2 | z_score ~ Age × Network + (1\|ID) | Age by network interaction | 79.772 | 0.999 | 0.393 | 0.094 | 0.330 | 0.233 | 0.263 |  |

**Model performance of different model sets for HbO**

| **Prenatal questions (HbO)** | | | | | | | | | |
| --- | --- | --- | --- | --- | --- | --- | --- | --- | --- |
| **Model** | **Formula** | **Description** | **AIC** | **AIC_wt** | **R2_conditional** | **R2_marginal** | **ICC** | **RMSE** | **Sigma** |
| M0 | z_score ~ 1 + (1\|ID) | Null model with random intercept only | 73.265 | 0.018 | 0.311 | 0 | 0.311 | 0.252 | 0.281 |
| M1 | z_score ~ Anxiety + Depression + (1\|ID) | Main effects of anxiety and depression | 75.717 | 0.005 | 0.33 | 0.021 | 0.316 | 0.251 | 0.281 |
| M2 | z_score ~ Anxiety + Depression + Network + (1\|ID) | Adds network as a main effect | 68.029 | 0.242 | 0.414 | 0.083 | 0.361 | 0.231 | 0.264 |
| M3 | z_score ~ Anxiety × Network + Depression × Network + (1\|ID) | Anxiety and depression by network interactions | 65.991 | 0.671 | 0.466 | 0.127 | 0.388 | 0.216 | 0.254 |
| M4 | z_score ~ Anxiety × Depression × Network + (1\|ID) | Three-way interaction among anxiety, depression, and network | 70.676 | 0.064 | 0.467 | 0.135 | 0.384 | 0.216 | 0.257 |

| **Postnatal questions (HbO)** | | | | | | | | | |
| --- | --- | --- | --- | --- | --- | --- | --- | --- | --- |
| **Model** | **Formula** | **Description** | **AIC** | **AIC_wt** | **R2_conditional** | **R2_marginal** | **ICC** | **RMSE** | **Sigma** |
| M0 | z_score ~ 1 + (1\|ID) | Null model with random intercept only | 85.953 | 0.001 | 0.242 | 0.000 | 0.242 | 0.277 | 0.303 |
| M1 | z_score ~ Anxiety + Depression + (1\|ID) | Main effects of anxiety and depression | 89.155 | 0.000 | 0.262 | 0.010 | 0.254 | 0.274 | 0.303 |
| M2 | z_score ~ Anxiety + Depression + Network + (1\|ID) | Adds network as a main effect | 72.964 | 0.964 | 0.418 | 0.119 | 0.338 | 0.238 | 0.271 |
| M3 | z_score ~ Anxiety × Network + Depression × Network + (1\|ID) | Anxiety and depression by network interactions | 79.823 | 0.031 | 0.407 | 0.122 | 0.324 | 0.238 | 0.276 |
| M4 | z_score ~ Anxiety × Depression × Network + (1\|ID) | Three-way interaction among anxiety, depression, and network | 84.217 | 0.003 | 0.416 | 0.127 | 0.332 | 0.235 | 0.277 |

| **Longitudinal questions (HbO)** | | | | | | | | | |
| --- | --- | --- | --- | --- | --- | --- | --- | --- | --- |
| **Model** | **Formula** | **Description** | **AIC** | **AIC_wt** | **R2_conditional** | **R2_marginal** | **ICC** | **RMSE** | **Sigma** |
| M0 | z_score ~ 1 + (1\|ID) | Null model with random intercept only | 63.959 | 0.013 | 0.313 | 0.000 | 0.313 | 0.267 | 0.298 |
| M1 | z_score ~ Δ_Anxiety + Δ_Depression + (1\|ID) | Main effects of symptom change in anxiety and depression | 66.300 | 0.004 | 0.340 | 0.031 | 0.319 | 0.265 | 0.298 |
| M2 | z_score ~ Δ_Anxiety + Δ_Depression + Prenatal_Anxiety + Prenatal_Depression + (1\|ID) | Adds prenatal baseline scores alongside symptom change | 69.831 | 0.001 | 0.367 | 0.041 | 0.340 | 0.262 | 0.298 |
| M3 | z_score ~ Δ_Anxiety:Prenatal_Anxiety + Δ_Depression:Prenatal_Depression + (1\|ID) | Tests interactions between symptom change and prenatal baseline only | 65.717 | 0.005 | 0.339 | 0.041 | 0.311 | 0.265 | 0.298 |
| M4 | z_score ~ Δ_Anxiety:Prenatal_Anxiety + Δ_Depression:Prenatal_Depression + Network + (1\|ID) | Adds network alongside change-by-baseline interactions | 59.738 | 0.107 | 0.434 | 0.112 | 0.363 | 0.241 | 0.277 |
| M5 | z_score ~ Network:Δ_Anxiety:Prenatal_Anxiety + Network:Δ_Depression:Prenatal_Depression + (1\|ID) | Three-way interactions: network, symptom change and prenatal baseline | 55.542 | 0.870 | 0.498 | 0.160 | 0.402 | 0.223 | 0.263 |

**Model performance of different model sets for HbR**

| **Testing age effect alone on functional connectivity (HbR)** | | | | | | | | | |
| --- | --- | --- | --- | --- | --- | --- | --- | --- | --- |
| **Model** | **Formula** | **Description** | **AIC** | **AIC_wt** | **R2_conditional** | **R2_marginal** | **ICC** | **RMSE** | **Sigma** |
| M0 | z_score ~ 1 + (1\|ID) | Null model with random intercept only | 84.9 | 0.004 | 0.204 | 0.000 | 0.204 | 0.264 | 0.286 |
| M1 | z_score ~ Age + (1\|ID) | Main effect of age on FC | 86.9 | 0.001 | 0.211 | 0.000 | 0.211 | 0.263 | 0.286 |
| M2 | z_score ~ Age × Network + (1\|ID) | Age by network interaction | 73.8 | 0.995 | 0.326 | 0.081 | 0.266 | 0.238 | 0.265 |

The model that includes the interaction between age and network outperforms both the null model and that with age as fixed effect, however no significant effect of age emerges and therefore age is not included as a predictor in the subsequent models.

| **Prenatal questions (HbR)** | | | | | | | | | |
| --- | --- | --- | --- | --- | --- | --- | --- | --- | --- |
| **Model** | **Formula** | **Description** | **AIC** | **AIC_wt** | **R2_conditional** | **R2_marginal** | **ICC** | **RMSE** | **Sigma** |
| M0 | z_score ~ 1 + (1\|ID) | Null model with random intercept only | 51.318 | 0.285 | 0.24 | 0 | 0.24 | 0.241 | 0.264 |
| M1 | z_score ~ Anxiety + Depression + (1\|ID) | Main effects of anxiety and depression | 54.543 | 0.057 | 0.26 | 0.01 | 0.252 | 0.239 | 0.264 |
| M2 | z_score ~ Anxiety + Depression + Network + (1\|ID) | Adds network as a main effect | 49.724 | 0.633 | 0.329 | 0.062 | 0.284 | 0.225 | 0.253 |
| M3 | z_score ~ Anxiety × Network + Depression × Network + (1\|ID) | Anxiety and depression by network interactions | 56.618 | 0.02 | 0.318 | 0.067 | 0.269 | 0.225 | 0.258 |
| M4 | z_score ~ Anxiety × Depression × Network + (1\|ID) | Three-way interaction among anxiety, depression, and network | 59.742 | 0.004 | 0.34 | 0.081 | 0.282 | 0.22 | 0.257 |

Main effect of network emerged (*F*(2,76) = 4.52, p = 0.01392)

| **Postnatal questions (HbR)** | | | | | | | | | |
| --- | --- | --- | --- | --- | --- | --- | --- | --- | --- |
| **Model** | **Formula** | **Description** | **AIC** | **AIC_wt** | **R2_conditional** | **R2_marginal** | **ICC** | **RMSE** | **Sigma** |
| M0 | z_score ~ 1 + (1\|ID) | Null model with random intercept only | 75.463 | 0.000 | 0.181 | 0.000 | 0.181 | 0.277 | 0.298 |
| M1 | z_score ~ Anxiety + Depression + (1\|ID) | Main effects of anxiety and depression | 78.019 | 0.000 | 0.199 | 0.016 | 0.186 | 0.275 | 0.298 |
| M2 | z_score ~ Anxiety + Depression + Network + (1\|ID) | Adds network as a main effect | 56.271 | 0.961 | 0.411 | 0.162 | 0.297 | 0.228 | 0.257 |
| M3 | z_score ~ Anxiety × Network + Depression × Network + (1\|ID) | Anxiety and depression by network interactions | 62.822 | 0.036 | 0.402 | 0.166 | 0.284 | 0.228 | 0.262 |
| M4 | z_score ~ Anxiety × Depression × Network + (1\|ID) | Three-way interaction among anxiety, depression, and network | 68.105 | 0.003 | 0.403 | 0.166 | 0.285 | 0.227 | 0.264 |

Main effect of network emerged (*F*(2,78) = 14.78, p < 0.001)

| **Longitudinal questions (HbR)** | | | | | | | | | | |
| --- | --- | --- | --- | --- | --- | --- | --- | --- | --- | --- |
| **Model** | **Formula** | **Description** | **AIC** | **AIC_wt** | **R2_conditional** | **R2_marginal** | **ICC** | **RMSE** | **Sigma** |  |
| M0 | z_score ~ 1 + (1\|ID) | Null model with random intercept only | 43.231 | 0.001 | 0.218 | 0.000 | 0.218 | 0.252 | 0.274 |  |
| M1 | z_score ~ Δ_Anxiety + Δ_Depression + (1\|ID) | Main effects of symptom change in anxiety and depression | 41.710 | 0.002 | 0.241 | 0.083 | 0.172 | 0.253 | 0.274 |  |
| M2 | z_score ~ Δ_Anxiety + Δ_Depression + Prenatal_Anxiety + Prenatal_Depression + (1\|ID) | Adds prenatal baseline scores alongside symptom change | 45.321 | 0.000 | 0.266 | 0.088 | 0.195 | 0.249 | 0.274 |  |
| M3 | z_score ~ Δ_Anxiety:Prenatal_Anxiety + Δ_Depression:Prenatal_Depression + (1\|ID) | Tests interactions between symptom change and prenatal baseline only | 41.578 | 0.002 | 0.241 | 0.084 | 0.170 | 0.253 | 0.274 |  |
| M4 | z_score ~ Δ_Anxiety:Prenatal_Anxiety + Δ_Depression:Prenatal_Depression + Network + (1\|ID) | Adds network alongside change-by-baseline interactions | 29.313 | 0.994 | 0.419 | 0.210 | 0.265 | 0.214 | 0.242 |  |
| M5 | z_score ~ Network:Δ_Anxiety:Prenatal_Anxiety + Network:Δ_Depression:Prenatal_Depression + (1\|ID) | Three-way interactions: network, symptom change and prenatal baseline | 46.615 | 0.000 | 0.251 | 0.106 | 0.162 | 0.250 | 0.278 |  |

Interaction effect of changes in depression score and their baseline at the prenatal timepoint emerged as significant (*F*(1,25) = 5.54, p = 0.026)

# 8. **Model Diagnostics**

Spearman’s rank correlations were run due to postnatal HADS subscales indicating non-normality (Shapiro-Wilk, p < .05). The results indicated a non-significant association between prenatal maternal anxiety and depression scores (*r*s(37) = .31, *p* = .058), and a statistically significant positive association between postnatal anxiety and depression scores (*r*s(38) = .51, *p* = .001).

**Prenatal Model (HbO)**

The selected prenatal model included an interaction between Anxiety and Network and Depression and Network. Adjusted variance inflation factors (GVIF^(1/(2·Df))) for our continuous predictors (Anxiety = 1.40; Depression = 1.40) indicated no problematic collinearity, while higher adjusted GVIFs for Network and its interaction terms (range: 2.17 – 2.25) were expected due to the categorical nature of predictors, although they remained below the conventional threshold of 3.16.

**Figure 8 SM.** Diagnostic plots for the selected prenatal HbO model. Model formula: z_score ~ Anxiety × Network + Depression × Network + (1 | ID).

**Postnatal Model (HbO)**

The selected postnatal model included main effects of Anxiety, Depression and Network. Adjusted variance inflation factors were examined and indicated no problematic collinearity (range = 1.00 – 1.104).

**Figure 9 SM.** Diagnostic plots for the selected postnatal HbO model. Model formula: z_score ~ Anxiety + Depression + Network + (1 | ID).

**Longitudinal Model (HbO)**

The selected longitudinal model included a three-way interaction between Network, Change in Anxiety and Prenatal Anxiety as well as a three-way interaction between Network, Change in Depression and Prenatal Depression. The adjusted variance inflation factors (GVIF^(1/(2·Df))) indicated no collinearity concerns between interaction terms (Network x Anxiety slope x Prenatal Anxiety = 1.003; Network x Depression slope x Prenatal Depression = 1.003).

**Figure 10 SM.** Diagnostic plots for the selected longitudinal HbO model. Model formula: z_score ~ Network:Δ_Anxiety:Prenatal_Anxiety + Network:Δ_Depression:Prenatal_Depression + (1 | ID).

**Prenatal Model (HbR)**

The selected prenatal model for HbR included main effects of Anxiety, Depression and Network. Adjusted variance inflation factors were examined and indicated no problematic collinearity (Anxiety = 1.079; Depression = 1.079, Network: 1.00).

**Figure 11 SM.** Diagnostic plots for the selected prenatal HbR model. Model formula: z_score ~ Anxiety + Depression + Network + (1|ID)

**Postnatal Model (HbR)**

The selected postnatal model for HbR included main effects of Anxiety, Depression and Network. Adjusted variance inflation factors indicated no problematic collinearity (Anxiety = 1.104; Depression = 1.104, Network: 1.00).

**Figure 12 SM.** Diagnostic plots for the selected postnatal HbR model. Model formula: z_score ~ Anxiety + Depression + Network + (1|ID)

**Longitudinal Model (HbR)**

The selected longitudinal model for HbR included main effects of Network and two-way interactions between Change in Anxiety and Prenatal Anxiety and between Change in Depression and Prenatal depression. The adjusted variance inflation factors (GVIF^(1/(2·Df))) indicated no collinearity concerns between predictors (Network = 1.000, Change in Anxiety x Prenatal Anxiety = 1.004; Change in Depression x Prenatal Depression = 1.004)

**Figure 13 SM.** Diagnostic plots for the selected longitudinal HbR model. Model formula: z_score ~ Δ_Anxiety:Prenatal_Anxiety + Δ_Depression:Prenatal_Depression + Network + (1|ID)

# 9. **Model Results for HbR**

The tables below include fixed effects results for the selected prenatal, postnatal and longitudinal models for HbR.

**Prenatal Model Results**

The model summary for the selected prenatal model for HbR revealed a significant main effect of network, with infants showing greater FC in the frontal network in comparison to the sensorimotor network (*β* = 0.13, 95% CI [0.02, 0.24], *t*(113) = 2.25, *p* = .027).

**Table 5 SM.**

*Full Model Results for the Selected Prenatal Model: HbR*

| **Term** | **β** | **SE** | **95% CI** | **t(110)** | **p** |
| --- | --- | --- | --- | --- | --- |
| **Fixed effects** | | | | | |
| (Intercept) | 0.40 | 0.08 | [0.23, 0.57] | 4.74 | < .001 |
| Anxiety | −0.007 | 0.010 | [−0.03, 0.01] | −0.71 | .482 |
| Depression | −0.003 | 0.010 | [−0.03, 0.02] | −0.24 | .812 |
| Network [Frontal] | 0.13 | 0.06 | [0.02, 0.24] | 2.25 | .027 |
| Network [Auditory] | −0.03 | 0.06 | [−0.15, 0.08] | −0.61 | .543 |
| **Random effects** | | | | | |
| Parameter | Coefficient |  |  |  |  |
| SD (Intercept: ID) | 0.16 | 0.04 | [0.10, 0.25] |  |  |
| SD (Residual) | 0.25 | 0.02 | [0.22, 0.30] |  |  |

**Note.** β = unstandardised regression coefficient. SE = standard error. 95% CIs and p-values were computed using a Wald t-distribution approximation. The reference level for Network is Sensorimotor. Model formula: z_score ~ Anxiety + Depression + Network + (1|ID)

**Postnatal Model Results**

The model summary for the selected postnatal model also revealed a significant main effect of network, with infants showing greater FC in the frontal network in comparison to the sensorimotor network (*β* = 0.27, 95% CI [0.15, 0.38], *t*(113) = 4.65, *p* < .001).

**Table 6 SM.**

*Full Model Results for the Selected Postnatal Model: HbR*

| Parameter | Coefficient | SE | 95% CI | t(113) | p |
| --- | --- | --- | --- | --- | --- |
| *Fixed effects* | | | | | |
| (Intercept) | 0.24 | 0.08 | [0.08, 0.40] | 3.03 | .003 |
| Anxiety | 0.003 | 0.01 | [−0.02, 0.02] | 0.27 | .786 |
| Depression | 0.01 | 0.01 | [−0.01, 0.03] | 0.94 | .350 |
| Network [Frontal] | 0.27 | 0.06 | [0.15, 0.38] | 4.65 | < .001 |
| Network [Auditory] | −0.007 | 0.06 | [−0.12, 0.11] | −0.12 | .908 |
| *Random effects* | | | | | |
| Parameter | Coefficient |  |  |  |  |
| SD (Intercept: ID) | 0.17 | 0.04 | [0.11, 0.26] |  |  |
| SD (Residual) | 0.26 | 0.02 | [0.22, 0.30] |  |  |

**Note.** β = unstandardised regression coefficient. SE = standard error. 95% CIs and p-values were computed using a Wald t-distribution approximation. The reference level for Network is Sensorimotor. Model formula: z_score ~ Anxiety + Depression + Network + (1|ID)

**Longitudinal Model Results**

A significant effect of network was observed in the frontal network relative to the sensorimotor network (*β* = 0.21, 95% CI [0.09, 0.34], *t*(77) = 3.31, *p* = .001). Furthermore, a significant interaction was also observed between depression slope and prenatal depression levels (*β* = 0.02, 95% CI [0.004, 0.05], *t*(77) = 2.35, *p* = .021), such that greater increases in maternal depression from prenatal to postnatal timepoints were associated with stronger HbR FC in the frontal network.

**Table 7 SM.**

*Full Model Results for the Selected Longitudinal Model: HbR*

| Parameter | Coefficient | SE | 95% CI | t(77) | p |
| --- | --- | --- | --- | --- | --- |
| *Fixed effects* | | | | | |
| (Intercept) | 0.32 | 0.05 | [0.21, 0.43] | 5.90 | < .001 |
| Network [Frontal] | 0.21 | 0.06 | [0.09, 0.34] | 3.31 | .001 |
| Network [Auditory] | −0.04 | 0.06 | [−0.17, 0.09] | −0.66 | .511 |
| Slope Anxiety × Prenatal Anxiety | −0.006 | 0.009 | [−0.02, 0.01] | −0.63 | .532 |
| Slope Depression × Prenatal Depression | 0.02 | 0.01 | [0.004, 0.05] | 2.35 | .021 |
| *Random effects* | | | | | |
| Parameter | Coefficient |  |  |  |  |
| SD (Intercept: ID) | 0.15 | 0.04 | [0.08, 0.25] |  |  |
| SD (Residual) | 0.24 | 0.02 | [0.20, 0.29] |  |  |

**Note.** β = unstandardised regression coefficient. SE = standard error. 95% CIs and p-values were computed using a Wald t-distribution approximation. The reference level for Network is Sensorimotor. Model formula: z_score ~ Δ_Anxiety:Prenatal_Anxiety + Δ_Depression:Prenatal_Depression + Network + (1|ID)

***Figure 14 SM.*** Group-level seed-based FC maps for the frontal (top), sensorimotor (middle) and auditory (bottom) networks. The maps show Fischer-Z transformed correlation values for each network, separately for left and right hemisphere seeds for HbR.

# **10. Significant Effects Across HbO and HbR**

Although we conducted primary analyses on HbO, the results, particularly for the postnatal models largely converged with HbR, as summarised in Table 8 SM. Across postnatal models, both HbO and HbR showed a significant effect of network, with greater FC in the frontal network relative to the sensorimotor network. Additionally, across all models and timepoints, no significant effects were found for anxiety. Some divergence was observed at the prenatal timepoint, with the selected HbO model including a significant effect of Network x Depression interaction, while the prenatal HbR model only showed a main effect of network. For the longitudinal models, the significant effects for both HbO and HbR indicated an interaction between change in depression from the prenatal to postnatal timepoint and greater FC in the frontal network, although for HbO the depression effect was more complex, with a three-way interaction with network, prenatal baseline, and change in symptoms.

**Table 8 SM.**

*Summary of Selected Models and Significant Effects for HbO and HbR Across Prenatal, Postnatal, and Longitudinal Timepoints*

| Timepoint | Signal | Selected Model | Significant Effect | β | 95% CI | Statistic | *p* |
| --- | --- | --- | --- | --- | --- | --- | --- |
| Prenatal | *HbO* | *z* ~ Anxiety × Network + Depression × Network + (1\|ID) | Network × Depression interaction, *F*(2, 72) = 4.16, *p* = .019 |  |  |  |  |
|  |  |  | Frontal network: higher prenatal depression → lower FC | −0.04 | [−0.09, −0.001] | *t*(106) = −2.02 | .046 |
|  | *HbR* | *z* ~ Anxiety + Depression + Network + (1\|ID) | Main effect of Network, *F*(2, 76) = 4.52, *p* = .014 |  |  |  |  |
|  |  |  | Frontal > Sensorimotor network | 0.13 | [0.02, 0.24] | *t*(113) = 2.25 | .027 |
| Postnatal | *HbO* | *z* ~ Anxiety + Depression + Network + (1\|ID) | Main effect of Network, *F*(2, 78) = 11.17, *p* < .001 |  |  |  |  |
|  |  |  | Frontal > Sensorimotor network | 0.24 | [0.12, 0.36] | *t*(113) = 3.92 | < .001 |
|  | *HbR* | *z* ~ Anxiety + Depression + Network + (1\|ID) | Main effect of Network, *F*(2, 78) = 14.78, *p* < .001 |  |  |  |  |
|  |  |  | Frontal > Sensorimotor network | 0.27 | [0.15, 0.38] | *t*(113) = 4.65 | < .001 |
| Longitudinal | *HbO* | *z* ~ Network:ΔAnxiety:Prenatal Anxiety + Network:ΔDepression:Prenatal Depression + (1\|ID) | Network × ΔDepression × Prenatal Depression, *F*(3, 37.94) = 6.12, *p* = .002 |  |  |  |  |
|  |  |  | Frontal network: greater increases in depression → stronger FC | 0.05 | [0.02, 0.09] | *t*(75) = 3.05 | .003 |
|  | *HbR* | *z* ~ ΔAnxiety:Prenatal Anxiety + ΔDepression:Prenatal Depression + Network + (1\|ID) | ΔDepression × Prenatal Depression interaction, *F*(1, 25) = 5.54, *p* = .026 |  |  |  |  |
|  |  |  | Frontal > Sensorimotor network | 0.21 | [0.09, 0.34] | *t*(77) = 3.31 | .001 |
|  |  |  | Greater increases in depression → stronger HbR FC in frontal network | 0.02 | [0.004, 0.05] | *t*(77) = 2.35 | .021 |

*Note.* HbO = oxyhaemoglobin; HbR = deoxyhaemoglobin; FC = functional connectivity; Δ = change (slope) from prenatal to postnatal timepoint. All models included participant (ID) as a random intercept. Reference network = Sensorimotor. *p*-values are two-tailed.
